# Supplementary figures and images for: Horizontal transfers between fungal Fusarium species contributed to successive outbreaks of coffee wilt disease
Source: PLoS Biol. 2024 Dec 5;22(12):e3002480. doi: 10.1371/journal.pbio.3002480 (PMC11620798; doi:10.1371/journal.pbio.3002480)

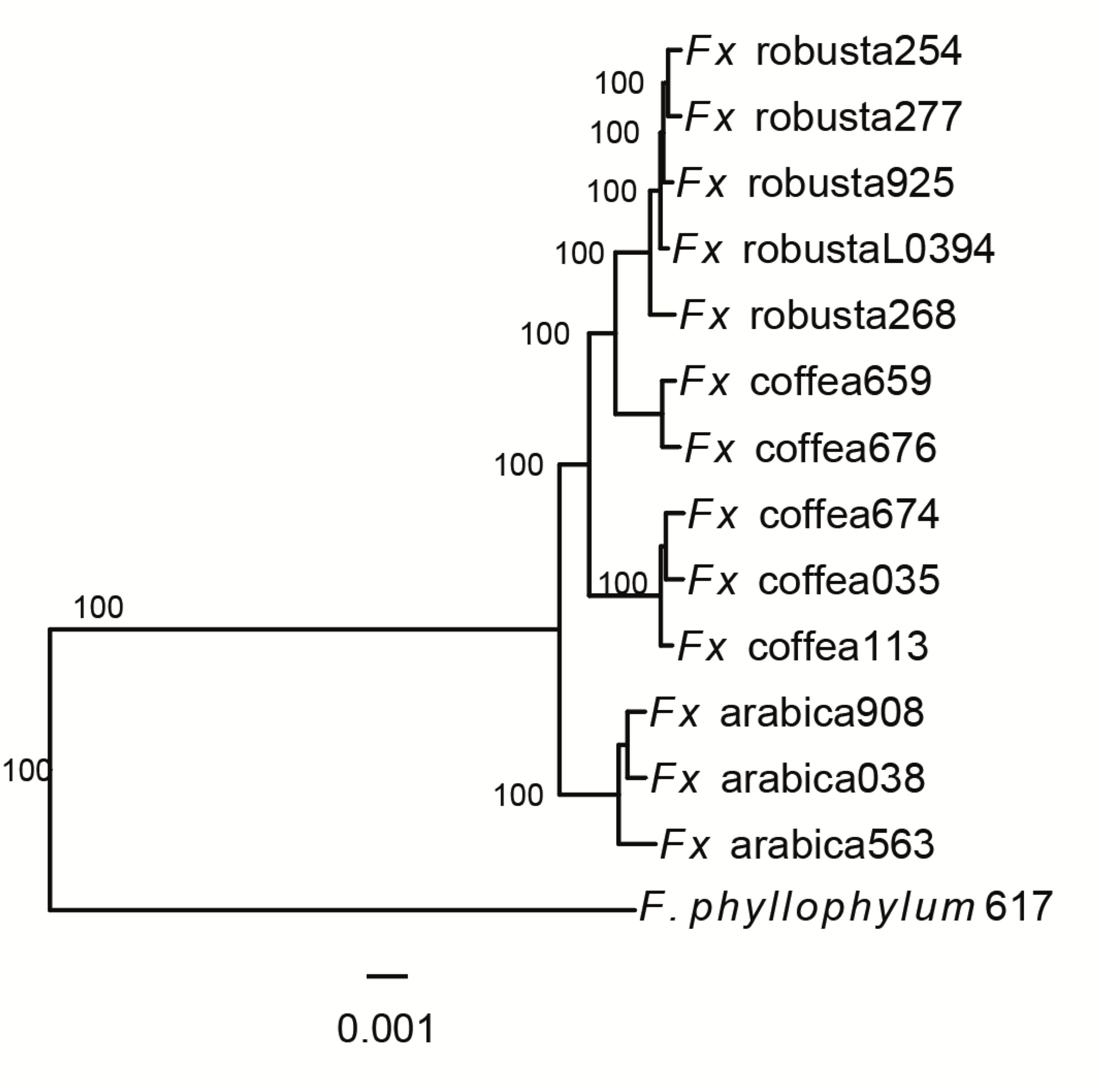

Supplement: S1 Fig — Annotated branches indicate bootstrap branch support values. The scale bar indicates branch length (0.001 substitutions per site). Fx, F. xylarioides. Full strain details in S1 and S2 Tables. The data underlying this figure can be found in https://doi.org/10.5281/zenodo.13836286. (TIFF) [file pbio.3002480.s001.tiff]

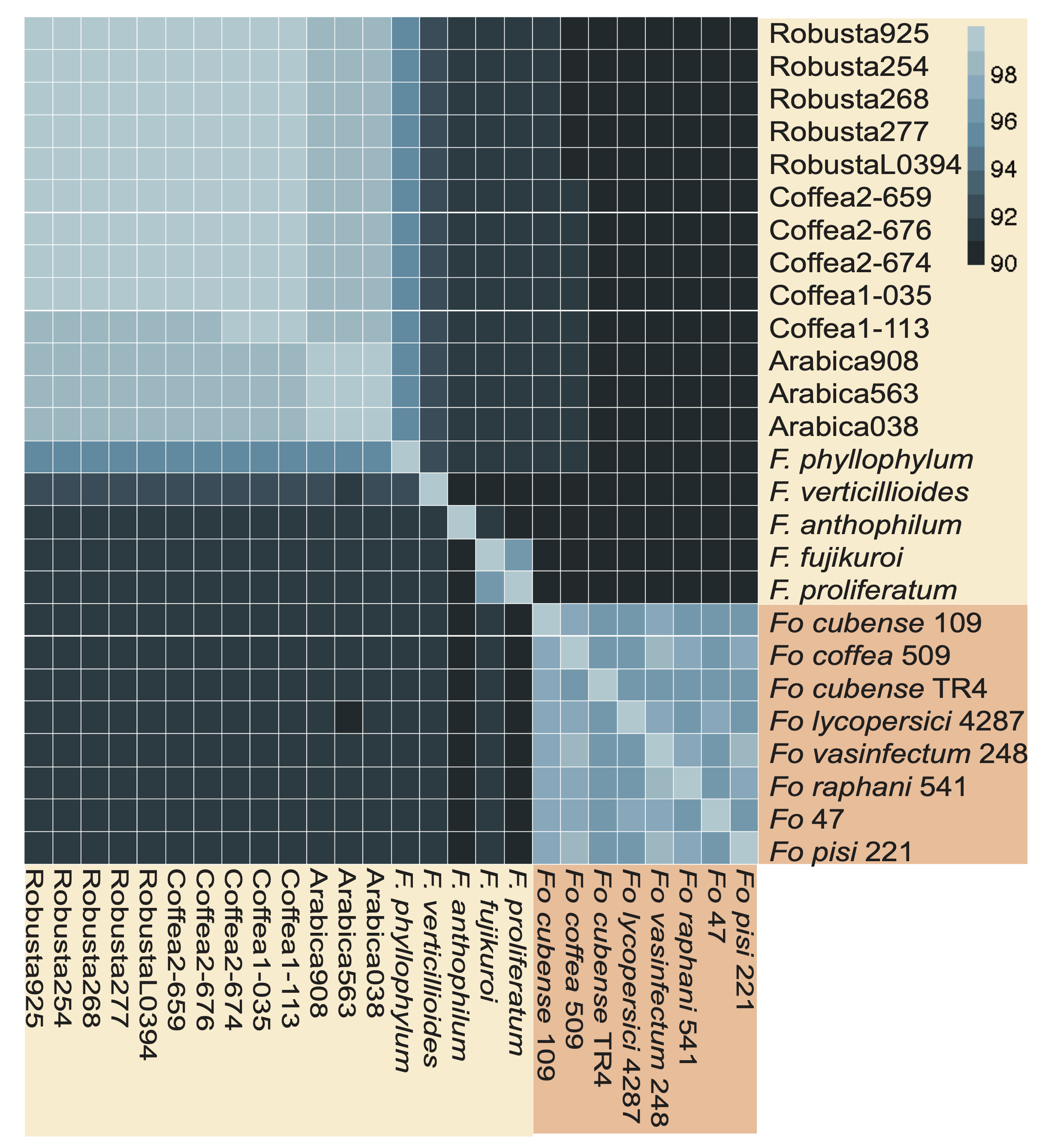

Supplement: S3 Fig — Cells indicate with colour the nucleotide similarity across all predicted coding sequences between the strains on the x and y axes. Shaded boxes refer to: the Fusarium oxysporum species complex, orange; and the Fusarium fujikuroi species complex, yellow, of which Fusarium xylarioides is a member. Arabica, robusta, coffea1, and coffea2 genomes all belong to Fusarium xylarioides. Excluding F. xylarioides, one genome is shown for each species/formae speciales with strain details in S2 Table. Fo, F. oxysporum. The data underlying this figure can be found in https://doi.org/10.5281/zenodo.13836286. (TIFF) [file pbio.3002480.s003.tiff]

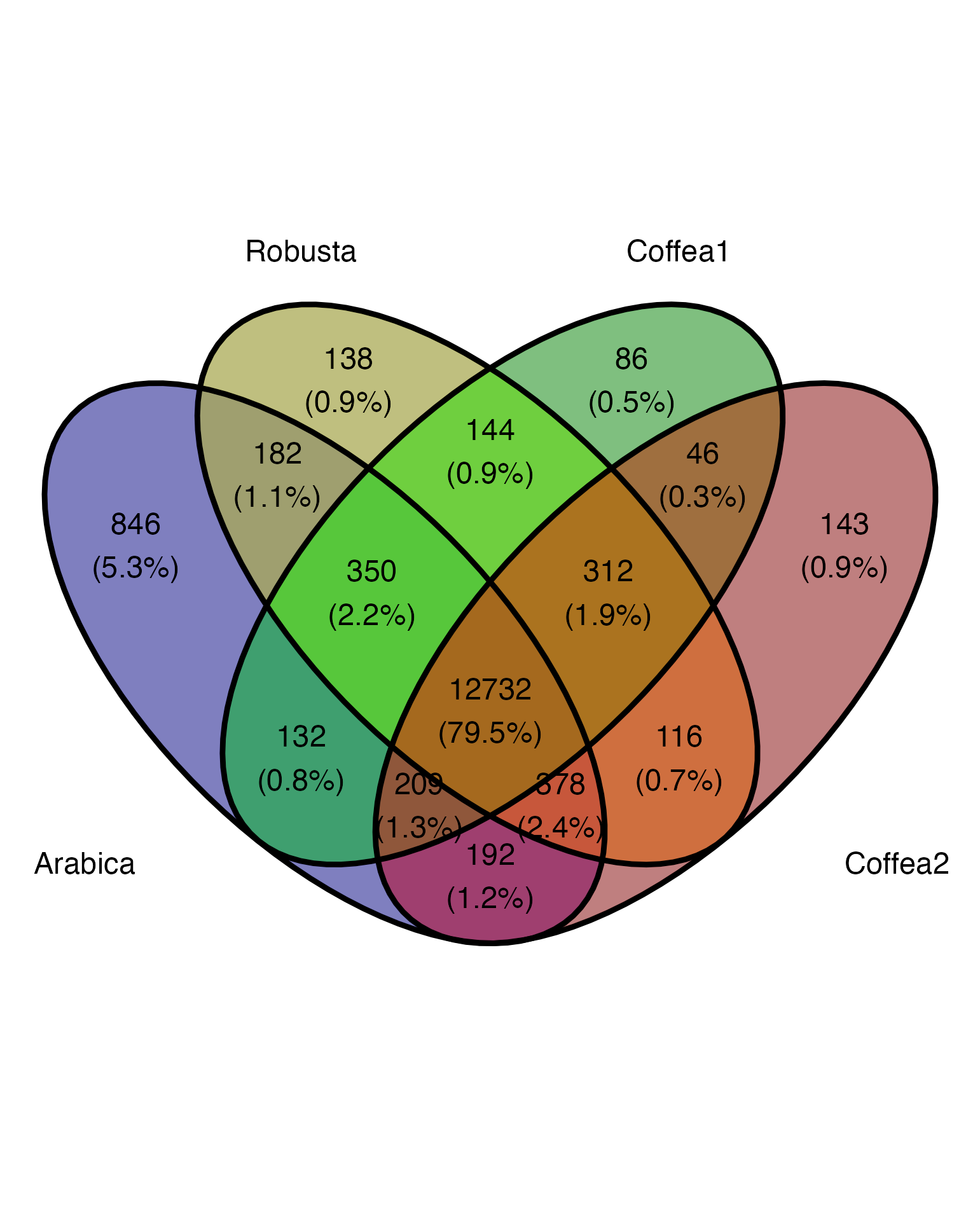

Supplement: S4 Fig — Orthogroups shared between F. xylarioides arabica, robusta, and the 2 coffea clusters. Drawn 16,006 (excluding 10,567 that were absent from F. xylarioides. (TIFF) [file pbio.3002480.s004.tiff]

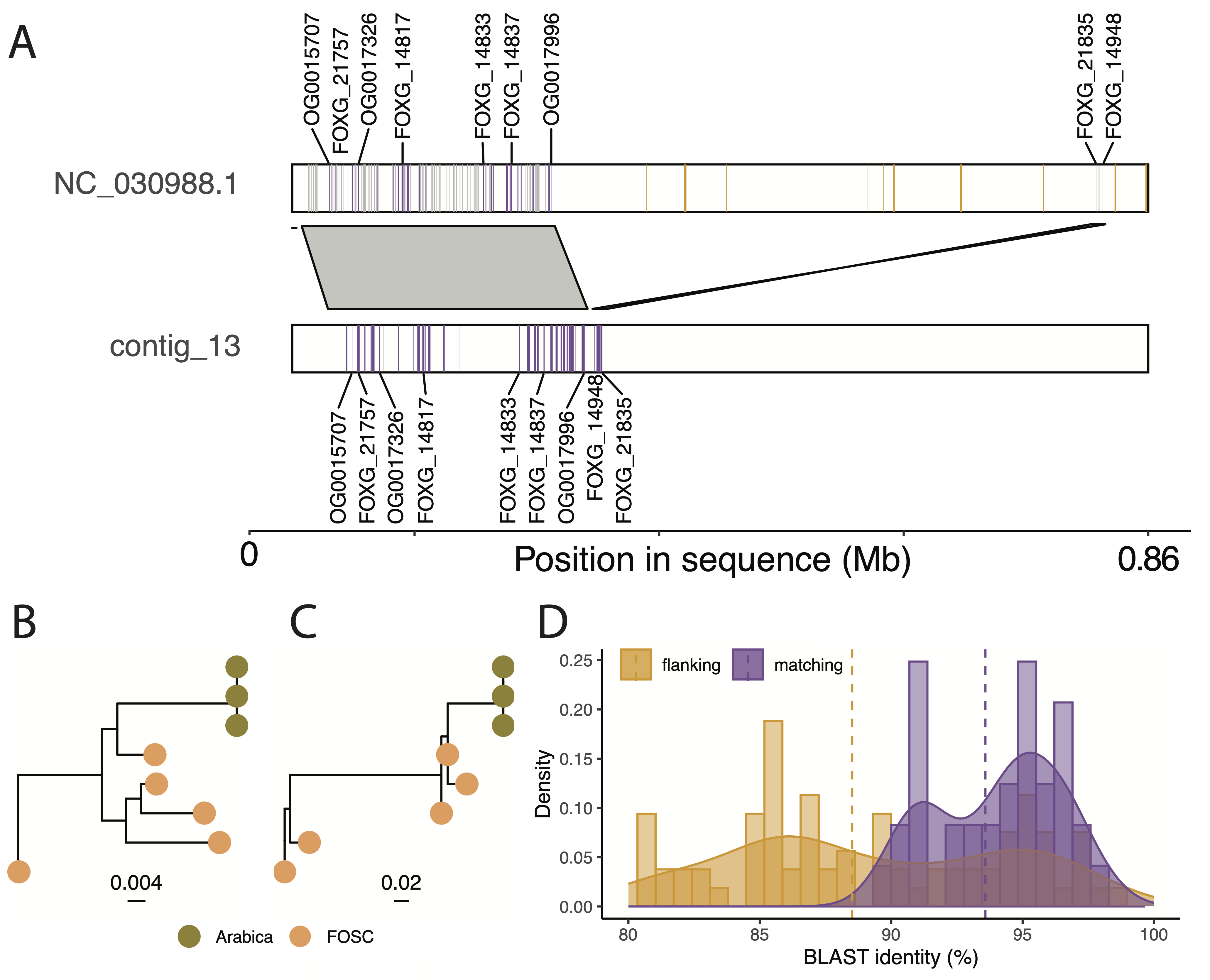

Supplement: S8 Fig — (A) HTR 1 on chromosome 1 in Fusarium xylarioides arabica563 (544 kb—739 kb, bottom plot) matches 2 regions in Fusarium oxysporum f. sp. lycopersici chromosome 3 (NC 030988, 4.7 Mb—5.6 Mb, top plot) in 2 pieces, large (200 kb) and small (20 kb). Highly similar genes shared by the 2 species are shaded in purple in top and bottom plots, a subset are labelled by Fusarium oxysporum gene locus tag, and non-matching genes found in the shared region are shaded grey. Genes flanking the shared region in Fusarium oxysporum are shaded yellow, and those which are found in Fusarium xylarioides (BLAST length >80%) have a lower sequence identity and are absent from contig 13. (B) A DendroBLAST rooted gene tree for the arabica563 gene H9Q71 0002169 (OG0015707) orthologous group, found in the shared region. (C) DendroBLAST gene tree for the arabica563 gene H9Q71 0003005 (OG0017996) orthologous group, found in the shared region. (D) The genes shared between the 2 species in the matching region have a higher BLAST sequence identity (mean 94%, dotted line) than those found in the flanking regions either side (mean 88%, dotted line). Figure drawn to scale. The data underlying this figure can be found in https://doi.org/10.5281/zenodo.13836286. (TIFF) [file pbio.3002480.s008.tiff]

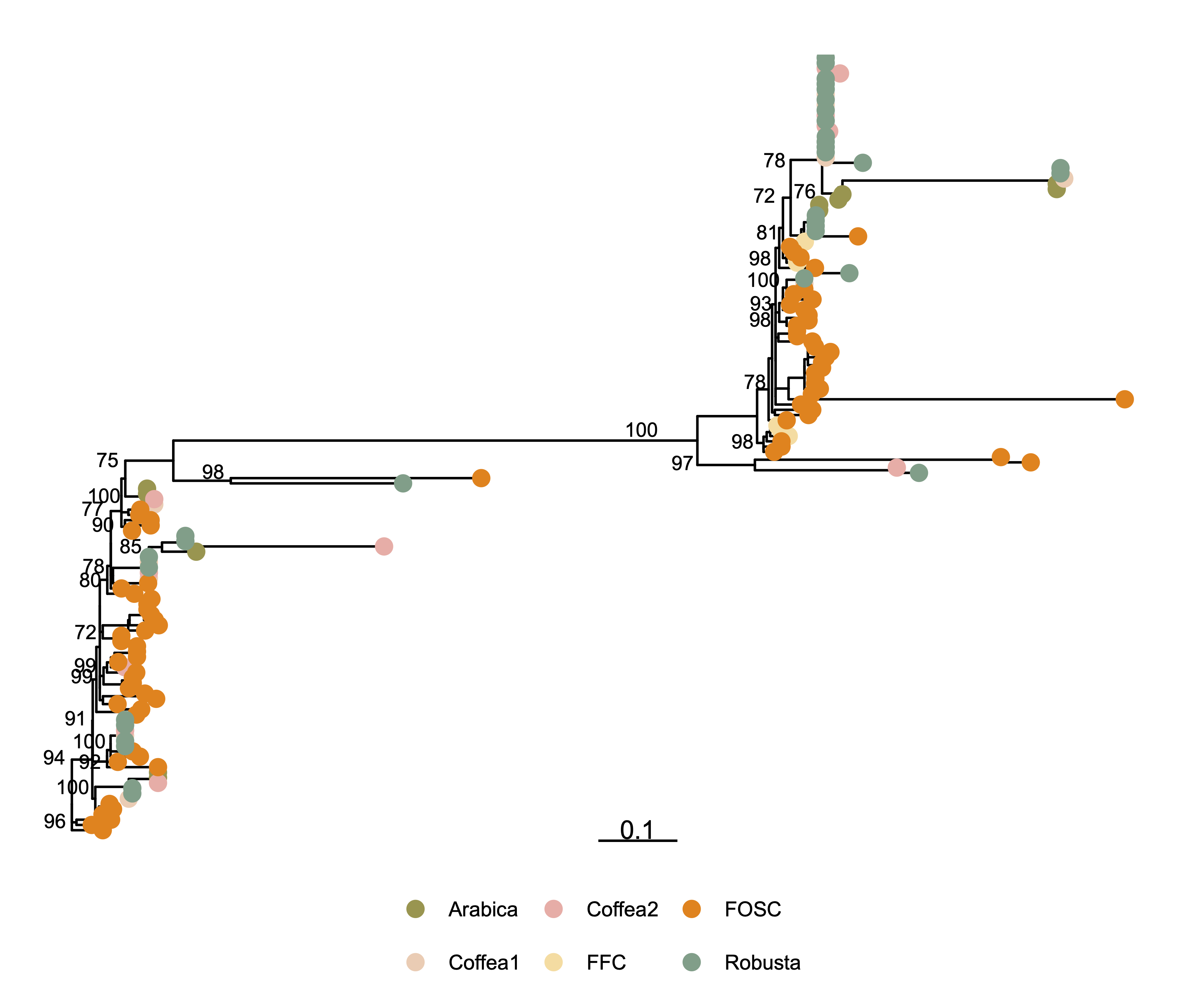

Supplement: S11 Fig — All mimp family 1 sequences from Fig 6 were aligned using MAFFT in Geneious v9.1.8. Using BLASTn, the consensus sequence was used to find copies in all other genomes used in this study (S2 and in the nr database. Any species with a hit >80% length and identity were selected (additional F. xylarioides and F. oxysporum hits were ignored), all sequences were re-aligned and a maximum likelihood tree with 1,000 bootstrap replicates constructed with IQ-TREE2. Bootstrap branch support is shown for all branches >0.7 (calculated with 1,000 bootstraps). Each point corresponds to a mimp sequence and the scalebar represents 0.1 substitutions per site. Tree tip labels are shaded by phylogenetic group: arabica, robusta, coffea1, and coffea2 F. xylarioides populations; FFC, F. fujikuroi species complex (all FFC tips are F. proliferatum; FOSC, F. oxysporum species complex. F. oxysporum strains are those annotated in this Fig 6). The data underlying this figure can be found in https://doi.org/10.5281/zenodo.13836286. (TIFF) [file pbio.3002480.s011.tiff]
