# Supplementary material for: Horizontal transfers between fungal Fusarium species contributed to successive outbreaks of coffee wilt disease
Source: PLoS Biol. 2024 Dec 5;22(12):e3002480. doi: 10.1371/journal.pbio.3002480 (PMC11620798; doi:10.1371/journal.pbio.3002480)
Supplement: S1 Table — (PDF) [file pbio.3002480.s012.pdf]

Table S1: **Bio-geographic details for strains sequenced in this study**

**Species**	**Short name**	**Strain**	**Year**	**Origin**	**Host**
F. xylarioides	Robusta268	392268	1968	DRC	*C. c. robusta*
F. xylarioides	Coffea676	392676	1964	Guinea?	*Coffea*
F. xylarioides	Coffea035	507035	1979/1963	Guinea	*C. canephora*
F. xylarioides	Coffea113	507113	1950	CAR	*unknown*
F. xylarioides	Arabica038	507038	1971	Ethiopia	*C. arabica*
F. oxysporum f. sp. raphani	*Fo raphani 541*	337541	1989	United States	*Raphanus sativus*
F. oxysporum	*Fo 509*	244509	1979	Tanzania	*C. arabica*
F. oxysporum f. sp. pisi	*Fo pisi 221*	500221	unknown	unknown	*Pisum sativum*
F. oxysporum f. sp. cubense	*Fo cubense 109*	141109	1969	United States	*Musa*
F. oxysporum f. sp. vasinfectum	*Fo vasinfectum 248*	292248	1985	Tanzania	*Gossypium*
F. solani	*F. solani 280*	392280	1963	Guinea	*C. c. robusta*
