# Supplementary material for: Horizontal transfers between fungal Fusarium species contributed to successive outbreaks of coffee wilt disease
Source: PLoS Biol. 2024 Dec 5;22(12):e3002480. doi: 10.1371/journal.pbio.3002480 (PMC11620798; doi:10.1371/journal.pbio.3002480)
Supplement: S2 Table — The columns describe: species, short species with strain number, accession number, whether the genome was used in OrthoFinder, whether the genome was used in S3B Fig for whole-genome similarity (Nsimscan). (PDF) [file pbio.3002480.s013.pdf]

Table S2: **Published genomes analysed in this study.** The columns describe: species, short species with strain number, accession number, whether the genome was used in OrthoFinder, whether the genome was used in Fig S3B for whole-genome similarity (Nsimscan).

Species	Short name	Published genomes	Orthofinder	Nsimscan
F. xylarioides 392674	Coffea674	GCA 018296325.1	Y	Y
F. xylarioides 127659i	Coffea659	GCA 018296285.1	Y	Y
F. xylarioides 392254	Robusta254	GCA 018296245.1	Y	Y
F. xylarioides 392277	Robusta277	GCA 018296265.1	Y	Y
F. xylarioides 375908i	Arabica908	GCA 018296345.1	Y	Y
F. xylarioides 389563	Arabica563	GCA 018296305.1	Y	Y
F. xylarioides L0394	RobustaL0394	GCA 013183765.1	Y	Y
F. xylarioides 379925	Robusta925	GCA 004329255.1	Y	Y
F. phyllophylum 617		GCA 013396025.1	Y	Y
F. udum 845		GCA 013186905.1	Y	Y
F. verticillioides 953		GCA 003316975.2	Y	
F. verticillioides 600		GCA 000149555.1	Y	Y
F. anthophyllum 214		GCA 013364935.1	Y	Y
F. proliferatum 237		GCA 017309865.1	Y	
F. proliferatum 157		GCA 900067095.1	Y	Y
F. fujikuroi LW94		GCA 001023035.1	Y	Y
F. fujikuroi Y057		GCA 001023045.1	Y	
F. oxysporum f. sp. raphani	*Fo raphani* Tf1262	GCA 019157275.1	Y	
F. oxysporum f. sp. raphani	*Fo raphani* 54005	GCA 000260235.2	Y	
F. oxysporum f. sp. pisi	*Fo pisi* HDV247	GCA 000260075.2	Y	
F. oxysporum f. sp. cubense	*Fo cubense* race1	GCA 000350345.1	Y	
F. oxysporum f. sp. vasinfectum	*Fo vasinfectum* 25433	GCA 000260175.2	Y	
F. oxysporum f. sp. cubense	*Fo cubense* b16	GCA 005930515.1	Y	
F. oxysporum f. sp. lycopersici	*Fo lycopersici* MN25	GCA 000259975.2	Y	
F. oxysporum 47	*Fo* 47	GCA 013085055.1	Y	Y
F. oxysporum f. sp. lycopersici	*Fo lycopersici* 4287	GCA 000149955.1	Y	Y
F. oxysporum f. sp. cubense	*Fo cubense* TR4	GCA 007994515.1	Y	Y
F. oxysporum f. sp. rapae	*Fo rapae* Tf1208	GCA 019157295.1	Y	
F. graminearum PH1		GCA 000240135.3	Y	Y
F. solani 657		GCA 020744495.1	Y	
F. oxysporum f. sp. *cubense*		[32]	Y	
