# Supplementary material for: Horizontal transfers between fungal Fusarium species contributed to successive outbreaks of coffee wilt disease
Source: PLoS Biol. 2024 Dec 5;22(12):e3002480. doi: 10.1371/journal.pbio.3002480 (PMC11620798; doi:10.1371/journal.pbio.3002480)
Supplement: S3 Table — Measured in 100 kb windows. (PDF) [file pbio.3002480.s014.pdf]

Table S3: Population genetics statistics related to genetic differentiation (d_{xy}) in four *Fusarium xylarioides* populations. Measured in 100 kb windows.

Nucleotide diversity (d_{xy})	Arabica	Robusta	Coffea1
Robusta	2.18E-03		
Coffea1	2.30E-03	1.78E-03	
Coffea2	2.29E-03	1.36E-03	1.71E-03
