# Supplementary material for: Horizontal transfers between fungal Fusarium species contributed to successive outbreaks of coffee wilt disease
Source: PLoS Biol. 2024 Dec 5;22(12):e3002480. doi: 10.1371/journal.pbio.3002480 (PMC11620798; doi:10.1371/journal.pbio.3002480)
Supplement: S4 Table — Species presence was determined using genealogies from OrthoFinder (see Methods) and BLAST similarity. A caret indicates a genealogy present in Fusarium udum, the other vascular wilt in the Fusarium fujikuroi species complex. Partial shading indicates partial presence across the population. An asterisk represents genealogies which match Fusarium oxysporum effector proteins. (PDF) [file pbio.3002480.s015.pdf]

Table S4: Nineteen up-regulated genes in both *Fusarium xylarioides* arabica strains are absent from the *Fusarium fujikuroi* complex (FFC) and differentially present across *Fusarium xylarioides*, the *Fusarium oxysporum* species complex (FOSC), the *Fusarium graminearum* species complex (FGC) and the *Fusarium solani* species complex (FSC). Species presence was determined using genealogies from OrthoFinder (see methods) and BLAST similarity. A caret indicates a genealogy present in *Fusarium udum*, the other vascular wilt in the *Fusarium fujikuroi* species complex. Partial shading indicates partial presence across the population. An asterisk represents genealogies which match *Fusarium oxysporum* effector proteins.

**Arabica563 gene**	**Phylogeny**							
	**Arabica**	**Robusta**	**Coffea1**	**Coffea2**	**FFC**	**FOSC**	**FGC**	**FSC**
H9Q71_0005697								
H9Q71_0004879								
H9Q71_0016000								
H9Q71_0001293								
H9Q71_0004347								
H9Q71_0004352								
H9Q71_0004355								
H9Q71_0011695								
H9Q71_0005751								
H9Q71_0017193						*		
H9Q71_0016558						*		
H9Q71_0017194						*		
H9Q71_0011583					^			
H9Q71_0015128								
H9Q71_0016978								
H9Q71_0013924								
H9Q71_0015127								
H9Q71_0011714								
H9Q71_0011713								
