# Supplementary material for: Horizontal transfers between fungal Fusarium species contributed to successive outbreaks of coffee wilt disease
Source: PLoS Biol. 2024 Dec 5;22(12):e3002480. doi: 10.1371/journal.pbio.3002480 (PMC11620798; doi:10.1371/journal.pbio.3002480)
Supplement: S5 Table — Only those which were up-regulated in both Fusarium xylarioides arabica563 and arabica908 are shown. The numbers in brackets describe the certainty. The orthogroup is shown for those predicted effectors up-regulated in both arabica563 and arabica908, and those which are annotated as carbohydrate-active enzymes are described. H9Q71 0010006 encodes an Ecp2 effector protein and H9Q71 0007533 contains a LysM domain. (PDF) [file pbio.3002480.s016.pdf]

Table S5: Predicted effectors in arabica563 genes by EffectorP [35]. Only those which were up-regulated in both *Fusarium xylarioides* arabica563 and arabica908 are shown. The numbers in brackets describe the certainty. The orthogroup is shown for those predicted effectors up-regulated in both arabica563 and arabica908, and those which are annotated as carbohydrate-active enzymes are described. H9Q71_0010006 encodes an Ecp2 effector protein and H9Q71_0007533 contains a LysM domain.

**Arabica563 gene**	**Cytoplasmic effector**	**Apoplastic effector**	**Prediction**	**Up-regulated in both arabica strains**	**CAZyme**
H9Q71_0003893	-	Y (0.536)	Apoplastic effector	OG0005463	PL9
H9Q71_0014967	-	Y (0.93)	Apoplastic effector	OG0000800	PL3
H9Q71_0001417	Y (0.648)	Y (0.945)	Apoplastic/cytoplasmic effector	OG0009694	PL3
H9Q71_0012305	Y (0.626)	-	Cytoplasmic effector	OG0005899	PL3
H9Q71_0006460	-	Y (0.658)	Apoplastic effector	OG0007961	PL1
H9Q71_0007385	-	Y (0.517)	Apoplastic effector	OG0007346	PL1
H9Q71_0012787	-	Y (0.537)	Apoplastic effector	OG0005885	PL1
H9Q71_0017136	-	Y (0.519)	Apoplastic effector	OG0013750	GH5
H9Q71_0003124	Y (0.515)	-	Cytoplasmic effector	OG0011177	GH43
H9Q71_0010493	-	Y (0.731)	Apoplastic effector	OG0011590	GH11
H9Q71_0012813	-	Y (0.976)	Apoplastic effector	OG0011334	GH11
H9Q71_0012684	-	Y (0.917)	Apoplastic effector	OG0002450	CE5
H9Q71_0016336	-	Y (0.811)	Apoplastic effector	OG0013204	CE5
H9Q71_0013065	-	Y (0.685)	Apoplastic effector	OG0007257	CE12
H9Q71_0005566	Y (0.839)	-	Cytoplasmic effector	OG0005610	CE12
H9Q71_0006667	-	Y (0.781)	Apoplastic effector	OG0003742	AA9
H9Q71_0013027	-	Y (0.671)	Apoplastic effector	OG0004566	AA9
H9Q71_0015352	-	Y (0.808)	Apoplastic effector	OG0004766	AA9
H9Q71_0000852	-	Y (0.957)	Apoplastic effector	OG0009750	
H9Q71_0002027	-	Y (0.622)	Apoplastic effector	OG0009728	
H9Q71_0002281	-	Y (0.917)	Apoplastic effector	OG0000381	
H9Q71_0003768	-	Y (0.731)	Apoplastic effector	OG0007484	
H9Q71_0010123	-	Y (0.924)	Apoplastic effector	OG0003872	
H9Q71_0016545	-	Y (0.681)	Apoplastic effector	OG0013167	
H9Q71_0017194	-	Y (0.734)	Apoplastic effector	OG0016169	
H9Q71_0001327	Y (0.789)	Y (0.868)	Apoplastic/cytoplasmic effector	OG0009809	
H9Q71_0006914	Y (0.512)	Y (0.83)	Apoplastic/cytoplasmic effector	OG0004608	
H9Q71_0010006	Y (0.562)	Y (0.719)	Apoplastic/cytoplasmic effector	OG0005427	
H9Q71_0013713	Y (0.567)	Y (0.661)	Apoplastic/cytoplasmic effector	OG0010581	
H9Q71_0017071	Y (0.58)	Y (0.765)	Apoplastic/cytoplasmic effector	OG0011892	
H9Q71_0000508	Y (0.843)	-	Cytoplasmic effector	OG0007573	
H9Q71_0001778	Y (0.542)	-	Cytoplasmic effector	OG0008003	
H9Q71_0001791	Y (0.835)	-	Cytoplasmic effector	OG0011563	
H9Q71_0003698	Y (0.685)	-	Cytoplasmic effector	OG0004761	
H9Q71_0005747	Y (0.644)	-	Cytoplasmic effector	OG0009835	
H9Q71_0008568	Y (0.613)	-	Cytoplasmic effector	OG0007844	
H9Q71_0009885	Y (0.6)	-	Cytoplasmic effector	OG0011803	
H9Q71_0015117	Y (0.675)	-	Cytoplasmic effector	OG0006788	
H9Q71_0007533	Y (0.685)	Y (0.64)	Cytoplasmic/apoplastic effector	OG0009487	
H9Q71_0012087	Y (0.734)	Y (0.638)	Cytoplasmic/apoplastic effector	OG0010978	
H9Q71_0012471	Y (0.604)	Y (0.547)	Cytoplasmic/apoplastic effector	OG0000156	
