# Supplementary material for: Horizontal transfers between fungal Fusarium species contributed to successive outbreaks of coffee wilt disease
Source: PLoS Biol. 2024 Dec 5;22(12):e3002480. doi: 10.1371/journal.pbio.3002480 (PMC11620798; doi:10.1371/journal.pbio.3002480)
Supplement: S6 Table — Each gene is described as up-regulated in planta (“coffee.up”), in axenic (“culture.up”), or not differentially expressed (“ns”). (PDF) [file pbio.3002480.s017.pdf]

Table S6: Expression data for all putative effectors from [29] across the *Fusarium xylarioides* arabica563 samples. Each gene is described as up-regulated *in planta* (“coffee.up”), *in axenic* (“culture.up”) or not differentially expressed (“ns”).

**Arabica563 gene**	**log2FoldChange**	**False Discovery Rate**	**Predicted EffectorP?**	**Differentially expressed?**	**Putative effector**
H9Q71_0011695-T1	13.4154519	7.18E-47	FALSE	coffee.up	OG0014398
H9Q71_0015569-T1	5.379337	3.84E-29	FALSE	coffee.up	FOXG_14254
H9Q71_0014967-T1	14.4073093	2.66E-16	TRUE	coffee.up	pelA
H9Q71_0004459-T1	11.736764	4.71E-16	TRUE	coffee.up	pelD
H9Q71_0002381-T1	2.5053746	1.88E-10	FALSE	coffee.up	orx1
H9Q71_0017001-T1	4.9013526	2.62E-09	FALSE	coffee.up	OG0018569
H9Q71_0007374-T1	-4.8076726	2.45E-07	FALSE	culture.up	OG0014367
H9Q71_0007948-T1	2.3398743	2.93E-06	FALSE	coffee.up	sge1
H9Q71_0014428-T1	3.9418829	7.27E-05	FALSE	coffee.up	catalase-peroxidase
H9Q71_0013555-T1	1.0310702	9.98E-04	FALSE	ns	fow1
H9Q71_0005049-T1	-0.7238584	4.23E-03	FALSE	ns	snf1
H9Q71_0012661-T1	-2.5494558	3.20E-02	FALSE	ns	chlo_vacu
H9Q71_0013807-T1	-2.0638231	5.75E-02	FALSE	ns	OG0014828
H9Q71_0001679-T1	-2.871467	1.09E-01	FALSE	ns	OG0013912
H9Q71_0002427-T1	0.3631168	1.23E-01	FALSE	ns	rho1.1
H9Q71_0003388-T1	-1.6549056	1.82E-01	FALSE	ns	OG0013871
H9Q71_0013972-T1	-0.2587035	4.35E-01	FALSE	ns	rho1.2
H9Q71_0016733-T1	-0.3777736	5.01E-01	FALSE	ns	OG0013877
H9Q71_0016313-T1	0.141086	8.53E-01	FALSE	ns	fmk1
H9Q71_0017133-T1	15.6417987	1.00E+00	TRUE	ns	OG0013477
