# Supplementary material for: Horizontal transfers between fungal Fusarium species contributed to successive outbreaks of coffee wilt disease
Source: PLoS Biol. 2024 Dec 5;22(12):e3002480. doi: 10.1371/journal.pbio.3002480 (PMC11620798; doi:10.1371/journal.pbio.3002480)
Supplement: S7 Table — Each gene is described as up-regulated in planta (“coffee.up”), in axenic (“culture.up”), or not differentially expressed (“ns”). (PDF) [file pbio.3002480.s018.pdf]

Table S7: Expression data for all putative effectors from [29] across the *Fusarium xylarioides* arabica908 samples. Each gene is described as up-regulated *in planta* (“coffee.up”), *in axenic* (“culture.up”) or not differentially expressed (“ns”).

| Arabica908 gene | log2FoldChange | False Discovery Rate | Predicted EffectorP? | Differentially expressed? | Putative effector |
|---|---|---|---|---|---|
| H9Q74_0007834-T1 | 13.65516535 | 2.00E-31 | FALSE | coffee.up | OG0014398 |
| H9Q74_0014203-T1 | 15.08200554 | 6.55E-21 | FALSE | coffee.up | OG0013477 |
| H9Q74_0011047-T1 | 11.68459215 | 2.55E-12 | TRUE | coffee.up | pelA |
| H9Q74_0013465-T1 | 3.95852869 | 2.95E-11 | FALSE | coffee.up | FOXG_14254 |
| H9Q74_0003935-T1 | 5.62411861 | 1.53E-08 | TRUE | coffee.up | pelD |
| H9Q74_0002710-T1 | -2.80463765 | 6.78E-07 | FALSE | culture.up | OG0014367 |
| H9Q74_014533-T1 | -2.44720929 | 5.95E-04 | FALSE | culture.up | snf1 |
| H9Q74_0001496-T1 | 1.96376611 | 1.62E-02 | FALSE | ns | sge1 |
| H9Q74_0010608-T1 | 0.74207488 | 1.98E-02 | FALSE | ns | fow1 |
| H9Q74_0002347-T1 | -2.46547337 | 3.34E-02 | FALSE | ns | catalase-peroxidase |
| H9Q74_0002576-T1 | -2.59379467 | 6.40E-02 | FALSE | ns | chlo_vacu |
| H9Q74_0012885-T1 | 1.72423883 | 9.40E-02 | FALSE | ns | OG0018569 |
| H9Q74_0012246-T1 | -1.08726 | 1.13E-01 | FALSE | ns | fmk1 |
| H9Q74_0005732-T1 | -0.46920318 | 1.54E-01 | FALSE | ns | rho1.2 |
| H9Q74_0001729-T1 | -0.35483043 | 4.68E-01 | FALSE | ns | orx1 |
| H9Q74_0010428-T1 | 0.6993708 | 5.53E-01 | FALSE | ns | OG0013871 |
| H9Q74_0013741-T1 | -0.10393013 | 8.78E-01 | FALSE | ns | OG0013877 |
| H9Q74_0001603-T1 | -0.04693784 | 9.18E-01 | FALSE | ns | rho1.1 |
