# Supplementary material for: Horizontal transfers between fungal Fusarium species contributed to successive outbreaks of coffee wilt disease
Source: PLoS Biol. 2024 Dec 5;22(12):e3002480. doi: 10.1371/journal.pbio.3002480 (PMC11620798; doi:10.1371/journal.pbio.3002480)
Supplement: S9 Table — The in planta up-regulated genes are shaded, where the warmest colours represent the highest up-regulated gene count. (PDF) [file pbio.3002480.s020.pdf]

Table S9: The number of genes expressed for each carbohydrate-active enzyme sub-family in *Fusarium xylarioides* arabica908. The *in planta* up-regulated genes are shaded, where the warmest colours represent the highest up-regulated gene count.

CAZyme	Gene annotation (n)	In planta up genes (n)	In axenic up genes (n)	Most differentially expressed gene (n)
AA1	13	0	3	0
AA11	4	0	3	0
AA12	2	0	0	0
AA13	1	0	0	0
AA14	1	0	0	0
AA16	1	0	0	0
AA2	4	0	1	0
AA3	24	3	1	0
AA3,AA8	0	0	0	0
AA4	5	0	1	0
AA4,AA4	0	0	0	0
AA4,AA7	0	0	0	0
AA5	1	0	0	0
AA5,CBM32	0	0	0	0
AA6	1	1	0	0
AA7	21	3	2	1
AA7,AA4	0	0	0	0
AA8,AA3	6	3	0	0
AA9	14	3	0	1
CBM18	2	0	0	0
CBM18,CE4	1	0	0	0
CBM18,GH18	1	0	0	0
CBM20,GH15	0	0	0	0
CBM21	1	0	0	0
CBM32,AA5	3	0	1	0
CBM38,GH32	0	0	0	0
CBM42,GH54	0	0	0	0
CBM43,GH72	0	0	0	0
CBM50	2	0	0	0
CBM6	0	0	0	0
CBM63	1	0	0	0
CBM67,GH78	0	0	0	0
CE1	4	1	0	0
CE10	55	0	1	0
CE10”,CE10”)	1	0	0	0
CE12	4	2	0	0
CE16	6	2	0	0
CE2	1	0	0	0
CE3	4	0	0	0
CE4	9	3	0	0

CAZyme	Gene annotation (n)	In planta up genes (n)	In axenic up genes (n)	Most differentially expressed gene (n)
CE5	12	3	0	1
CE7	1	0	0	0
CE8	4	1	0	0
CE9	1	0	0	0
GH1	4	0	0	0
GH10	4	2	0	1
GH105	4	3	0	1
GH106	1	0	0	0
GH11	3	2	0	0
GH114	3	0	1	0
GH115	2	0	0	0
GH12	4	0	1	0
GH125	3	1	0	0
GH127,GH146	0	0	0	0
GH128	3	1	0	0
GH13	8	1	0	0
GH131	1	0	0	0
GH132	1	0	0	0
GH133	1	0	0	0
GH134	1	0	0	0
GH139	1	0	0	0
GH145	2	0	0	0
GH145,PL24	0	0	0	0
GH146	1	0	0	0
GH146,GH127	1	1	0	0
GH146,GH127,GH146,GH127	0	0	0	0
GH15	1	0	0	0
GH15,CBM20	1	0	0	0
GH152	1	0	0	0
GH154	2	0	0	0
GH16	21	1	2	0
GH16,GH64	0	0	0	0
GH162	1	0	0	0
GH17	4	0	0	0
GH18	17	2	2	0
GH2	9	3	0	0
GH20	3	0	2	0
GH24	1	0	0	0
GH28	8	2	0	0
GH28,GH28	0	0	0	0
GH29	3	1	0	0
GH3	19	2	1	1
GH30	2	0	0	0
GH31	8	1	0	0

CAZyme	Gene annotation (n)	In planta up genes (n)	In axenic up genes (n)	Most differentially expressed gene (n)
GH32	7	1	0	0
GH32,CBM38	2	0	0	0
GH33	1	0	0	0
GH35	6	1	0	0
GH36	3	1	0	0
GH37	2	1	0	0
GH38	0	0	0	0
GH43	23	13	0	2
GH43,CBM6	1	0	0	0
GH45	1	0	0	0
GH47	10	1	1	0
GH49	1	0	0	0
GH5	18	3	0	1
GH5,GH2	0	0	0	0
GH51	2	1	0	0
GH53	1	0	0	0
GH54,CBM42	1	0	0	0
GH55	3	0	1	0
GH6	1	0	0	0
GH64	3	0	0	0
GH65	1	0	0	0
GH67	2	0	0	0
GH7	2	0	0	0
GH71	2	0	0	0
GH72	2	0	0	0
GH72,CBM43	1	0	0	0
GH75	1	0	0	0
GH76	11	0	1	0
GH78	2	0	0	0
GH78,CBM67	5	1	0	1
GH79	0	0	0	0
GH81	2	1	0	0
GH88	1	0	0	0
GH93	5	2	0	0
GH95	2	0	0	0
GT1	5	0	0	0
GT15	5	0	0	0
GT17	2	0	0	0
GT2_Chitin_synth	9	0	1	0
GT2_Glyco_tranf_2	1	0	1	0
GT2_Glyco_trans_2	3	0	0	0
GT2_Glycos_transf	3	0	0	0

CAZyme	Gene annotation (n)	In planta up genes (n)	In axenic up genes (n)	Most differentially expressed gene (n)
GT20	2	0	0	0
GT21	1	0	0	0
GT22	5	0	0	0
GT24	1	0	0	0
GT3	1	0	1	0
GT32	5	0	1	0
GT33	1	0	0	0
GT34	3	0	0	0
GT35	1	0	0	0
GT39	3	0	0	0
GT4	5	0	0	0
GT48	1	0	0	0
GT48,GT48”)	0	0	0	0
GT57	2	0	0	0
GT58	1	0	0	0
GT59	1	0	0	0
GT62	3	0	1	0
GT64	2	0	0	0
GT66	1	0	0	0
GT69	1	0	1	0
GT71	2	0	0	0
GT76	1	0	0	0
GT8	6	0	0	0
GT90	4	0	0	0
PL1	13	9	0	4
PL26	1	1	0	1
PL3	5	4	0	1
PL4	3	1	0	1
PL9	1	1	0	1
