# Supplementary material for: Horizontal transfers between fungal Fusarium species contributed to successive outbreaks of coffee wilt disease
Source: PLoS Biol. 2024 Dec 5;22(12):e3002480. doi: 10.1371/journal.pbio.3002480 (PMC11620798; doi:10.1371/journal.pbio.3002480)
Supplement: S10 Table — (PDF) [file pbio.3002480.s021.pdf]

Table S10: Gene count and description for significantly enriched InterPro terms in the *Coffea arabica in planta* samples

| **InterPro category** | ***Coffea arabica* genes** | **Gene number** | **Gene Ontology term** | **InterPro description** | **Pfam domain** |
|---|---|---|---|---|---|
| IPR002068 | XP_027098388.1, XP_027113320.1, XP_027095883.1, XP_027096450.1, XP_027109657.1 | 5 | NA | Hsp20/alpha crystallin family | PF00011 |
| IPR001938 | XP_027095536.1, XP_027096550.1, XP_027080774.1, XP_027080772.1 | 4 | NA | Thaumatin family | PF00314 |
| IPR007117 | XP_027083877.1, XP_027082625.1, XP_027083872.1, XP_027084129.1 | 4 | NA | Expansin C-terminal domain | PF01357 |
| IPR009009 | XP_027083877.1, XP_027082625.1, XP_027083872.1, XP_027084129.1 | 4 | NA | Lytic transglycolase | PF03330 |
| IPR013126 | XP_027125306.1, XP_027121874.1, XP_027122715.1, XP_027114671.1 | 4 | GO:0005524, GO:0140662 | Hsp70 protein | PF00012 |
| IPR001128 | XP_027119443.1, XP_027099114.1 | 2 | GO:0004497, GO:0005506, GO:0016705, GO:0020037 | Cytochrome P450 | PF00067 |
| IPR001155 | XP_027087183.1, XP_027088006.1 | 2 | GO:0010181, GO:0016491 | NADH:flavin oxidoreductase / NADH oxidase family | PF00724 |
| IPR001480 | XP_027061914.1, XP_027077059.1 | 2 | NA | D-mannose binding lectin | PF01453 |
| IPR002213 | XP_027098144.1, XP_027127315.1 | 2 | GO:0008194 | UDP-glucoronosyl and UDP-glucosyl transferase | PF00201 |

| InterPro category | *Coffea arabica* genes | Gene number | Gene Ontology term | InterPro description | Pfam domain |
|---|---|---|---|---|---|
| IPR003959 | XP_027077924.1, XP_027113909.1 | 2 | GO:0005524, GO:0016887 | ATPase family associated with various cellular activities (AAA) | PF00004 |
| IPR004265 | XP_027062967.1, XP_027062970.1 | 2 | NA | Dirigent-like protein | PF03018 |
| IPR008146 | XP_027077249.1, XP_027073453.1 | 2 | GO:0004356, GO:0006807 | Glutamine synthetase, catalytic domain | PF00120 |
| IPR008147 | XP_027077249.1, XP_027073453.1 | 2 | GO:0004356, GO:0006542, GO:0006807 | Glutamine synthetase, beta-Grasp domain | PF03951 |
| IPR008914 | XP_027068654.1, XP_027069261.1 | 2 | NA | Phosphatidylethanolamine-binding protein | PF01161 |
| IPR013148 | XP_027080458.1, XP_027072817.1 | 2 | NA | Glycosyl hydrolases family 32 N-terminal domain | PF00251 |
| IPR013189 | XP_027080458.1, XP_027072817.1 | 2 | NA | Glycosyl hydrolases family 32 C terminal | PF08244 |
| IPR000023 | XP_027062920.1 | 1 | GO:0003872, GO:0006096 | Phosphofructokinase | PF00365 |
| IPR000070 | XP_027113015.1 | 1 | GO:0030599, GO:0042545 | Pectinesterase | PF01095 |
| IPR000073 | XP_027107249.1 | 1 | NA | Alpha/beta hydrolase family | PF12697 |
| IPR000408 | XP_027070596.1 | 1 | NA | Regulator of chromosome condensation (RCC1) repeat | PF00415 |
| IPR000490 | XP_027067208.1 | 1 | GO:0004553, GO:0005975 | Glycosyl hydrolases family 17 | PF00332 |
| IPR000642 | XP_027077924.1 | 1 | GO:0004176, GO:0004222, GO:0005524, GO:0006508 | Peptidase family M41 | PF01434 |
| IPR000719 | XP_027077059.1 | 1 | GO:0004672, GO:0005524, GO:0006468 | Protein kinase domain | PF00069 |
| IPR000726 | XP_027075439.1 | 1 | GO:0004568, GO:0006032, GO:0016998 | Chitinase class I | PF00182 |

| InterPro category | *Coffea arabica* genes | Gene number | Gene Ontology term | InterPro description | Pfam domain |
|---|---|---|---|---|---|
| IPR000743 | XP_027119275.1 | 1 | GO:0004650, GO:0005975 | Glycosyl hydrolases family 28 | PF00295 |
| IPR000863 | XP_027095476.1 | 1 | GO:0008146 | Sulfotransferase domain | PF00685 |
| IPR000996 | XP_027064885.1 | 1 | GO:0005198, GO:0006886, GO:0016192, GO:0030130, GO:0030132 | Clathrin light chain | PF01086 |
| IPR001153 | XP_027109675.1 | 1 | GO:0042742, GO:0050832 | Barwin family | PF00967 |
| IPR001382 | XP_027123559.1 | 1 | GO:0004571, GO:0005509, GO:0016020 | Glycosyl hydrolase family 47 | PF01532 |
| IPR001404 | XP_027110216.1 | 1 | GO:0005524, GO:0006457, GO:0016887, GO:0051082, GO:0140662 | Hsp90 protein | PF00183 |
| IPR001611 | XP_027127483.1 | 1 | GO:0005515 | Leucine rich repeat | PF13855 |
| IPR001764 | XP_027124556.1 | 1 | GO:0004553, GO:0005975 | Glycosyl hydrolase family 3 N terminal domain | PF00933 |
| IPR002241 | XP_027100553.1 | 1 | GO:0004553, GO:0005975 | Alpha galactosidase A | PF16499 |
| IPR002347 | XP_027070209.1 | 1 | NA | short chain dehydrogenase | PF00106 |
| IPR002772 | XP_027124556.1 | 1 | GO:0004553, GO:0005975 | Glycosyl hydrolase family 3 C-terminal domain | PF01915 |
| IPR002912 | XP_027097796.1 | 1 | NA | ACT domain | PF01842 |
| IPR003245 | XP_027069566.1 | 1 | GO:0009055 | Plastocyanin-like domain | PF02298 |
| IPR003594 | XP_027110216.1 | 1 | NA | Histidine kinase-, DNA gyrase B-, and HSP90-like ATPase | PF02518 |
| IPR003609 | XP_027061914.1 | 1 | NA | PAN-like domain | PF08276 |
| IPR004240 | XP_027084986.1 | 1 | GO:0016021 | Endomembrane protein 70 | PF02990 |
| IPR004316 | XP_027100714.1 | 1 | GO:0016021 | Sugar efflux transporter for intercellular exchange | PF03083 |
| IPR004839 | XP_027112899.1 | 1 | GO:0009058, GO:0030170 | Aminotransferase class I and II | PF00155 |

| InterPro category | *Coffea arabica* genes | Gene number | Gene Ontology term | InterPro description | Pfam domain |
|---|---|---|---|---|---|
| IPR004843 | XP_027070343.1 | 1 | GO:0016787 | Calcineurin-like phosphoesterase | PF00149 |
| IPR005474 | XP_027102624.1 | 1 | NA | Transketolase, thiamine diphosphate binding domain | PF00456 |
| IPR005475 | XP_027102624.1 | 1 | NA | Transketolase, pyrimidine binding domain | PF02779 |
| IPR005828 | XP_027103991.1 | 1 | GO:0016021, GO:0022857, GO:0055085 | Sugar (and other) transporter | PF00083 |
| IPR006094 | XP_027088393.1 | 1 | GO:0050660 | FAD binding domain | PF01565 |
| IPR006254 | XP_027062002.1 | 1 | GO:0004451, GO:0019752 | Isocitrate lyase family | PF00463 |
| IPR006501 | XP_027113015.1 | 1 | GO:0004857 | Plant invertase/pectin methylesterase inhibitor | PF04043 |
| IPR012951 | XP_027088393.1 | 1 | GO:0016491, GO:0050660 | Berberine and berberine like | PF08031 |
| IPR013149 | XP_027120066.1 | 1 | NA | Zinc-binding dehydrogenase | PF00107 |
| IPR015914 | XP_027070343.1 | 1 | GO:0003993, GO:0046872 | Purple acid Phosphatase, N-terminal domain | PF16656 |
| IPR019378 | XP_027070430.1 | 1 | NA | GDP-fucose protein O-fucosyltransferase | PF10250 |
| IPR019791 | XP_027102149.1 | 1 | NA | Animal haem peroxidase | PF03098 |
| IPR022003 | XP_027086201.1 | 1 | NA | RCD1-SRO-TAF4 (RST) plant domain | PF12174 |
| IPR022702 | XP_027127341.1 | 1 | NA | Cytosine specific DNA methyltransferase replication foci domain | PF12047 |
| IPR025733 | XP_027070343.1 | 1 | NA | Iron/zinc purple acid phosphatase-like protein C | PF14008 |
| IPR026992 | XP_027116487.1 | 1 | NA | non-haem dioxygenase in morphine synthesis N-terminal | PF14226 |
| IPR033248 | XP_027102624.1 | 1 | NA | Transketolase, C-terminal domain | PF02780 |
| IPR041233 | XP_027100553.1 | 1 | NA | Alpha galactosidase C-terminal beta sandwich domain | PF17801 |
| IPR041569 | XP_027077924.1 | 1 | NA | AAA+ lid domain | PF17862 |
| IPR041694 | XP_027120066.1 | 1 | NA | N-terminal domain of oxidoreductase | PF16884 |
| IPR044861 | XP_027116487.1 | 1 | NA | 2OG-Fe(II) oxygenase superfamily | PF03171 |
