# Supplementary material for: Horizontal transfers between fungal Fusarium species contributed to successive outbreaks of coffee wilt disease
Source: PLoS Biol. 2024 Dec 5;22(12):e3002480. doi: 10.1371/journal.pbio.3002480 (PMC11620798; doi:10.1371/journal.pbio.3002480)
Supplement: S11 Table — Low complexity and simple repeats were excluded from the transposable element (TE) count. “TE of interest” refers to those transposable elements identified on the F. oxysporum mobile pathogenic chromsome in [25] and the unclassified TE “rnd-6 family-1942.” sc2, supercontig 2. (PDF) [file pbio.3002480.s022.pdf]

Table S11: Transposable element density in horizontally transferred regions (HTR), *Starships* and genome-wide in the *Fusarium xylarioides* arabica563 reference. Low complexity and simple repeats were excluded from the transposable element (TE) count. ”TE of interest” refers to those transposable elements identified on the *F. oxysporum* mobile pathogenic chromsome in [26] and the unclassified TE ”rnd-6_family-1942”. Abbreviations: sc2, supercontig 2.

HTR	TE count	TE/bp	TE of interest count
HTR 1	155	0.078	36
HTR 2	62	0.207	10
HTR 3	51	0.102	0
HTR 4	68	0.082	0
HTR 5	100	0.111	19
HTR 6	23	0.115	16
Genome average	7.6	0.076	132
