# Supplementary material for: Horizontal transfers between fungal Fusarium species contributed to successive outbreaks of coffee wilt disease
Source: PLoS Biol. 2024 Dec 5;22(12):e3002480. doi: 10.1371/journal.pbio.3002480 (PMC11620798; doi:10.1371/journal.pbio.3002480)
Supplement: S12 Table — The second column represents the contigs as named in the public assembly under accession PRJNA1043203. In the final column, each contig has been renamed to first correspond to its core Fusarium chromosome, and then its order in the whole genome visualisation (Fig 1A). (PDF) [file pbio.3002480.s023.pdf]

Table S12: The contigs in the *Fusarium xylarioides* arabica563 reference genome which correspond to each chromosome in the *F. verticillioides* assembly [22]. The second column represents the contigs as named in the public assembly under accession PRJNA1043203. In the final column, each contig has been renamed to first correspond to its core *Fusarium* chromosome; and then its order in the whole genome visualisation (Fig 1A).

Fusarium verticillioides chromosome	Reference arabica563 contig	Reference arabica563 corresponding chromosome
1	13	1.1
1	1	1.2
2	2	2
3	4	3
5	3	5
6	12	6.1
6	15	6.2
6	14	6.3
6	8	6.4
8	7	8
9	9	9
10	10	10
11	11	11
4 & 7	5	sc1
4 & 7	6	sc2
