# Supplementary material for: Horizontal transfers between fungal Fusarium species contributed to successive outbreaks of coffee wilt disease
Source: PLoS Biol. 2024 Dec 5;22(12):e3002480. doi: 10.1371/journal.pbio.3002480 (PMC11620798; doi:10.1371/journal.pbio.3002480)
Supplement: S13 Table — Mean bedtools coverage in 10 kb regions relative to the whole genome. I_Fx = mean similarity with other F. xylarioides populations across 10 kb windows within HTR. I_Fo = mean similarity with closest F. oxysporum match across 10 kb windows within HTR. I_Diff is I_Fx - I_Fo, a metric of how similar is F. xylarioides versus F. oxysporum: positive value = more similar to F. xylarioides; negative = more similar to F. oxysporum. p = proportion of values of I_Diff from all contiguous regions of genome the same size as HTR that are ≤ the observed value of I_Diff for the HTR. (PDF) [file pbio.3002480.s024.pdf]

Table S13: Comparison of divergence patterns for HTR regions to those of background genome regions. Mean bedtools coverage in 10Kb regions relative to the whole genome.
I_Fx = mean similarity with other *F. xylarioides* populations across 10 kb windows within HTR.
I_Fo = mean similarity with closest *F. oxysporum* match across 10 kb windows within HTR.
I_diff is *I_Fx* - *I_Fo*, a metric of how similar is *F. xylarioides* versus *F. oxysporum*: positive value = more similar to *F. xylarioides*; negative = more similar to *F. oxysporum*.
p = proportion of values of *I_diff* from all contiguous regions of genome the same size as HTR that are <= the observed value of *I_diff* for the HTR.

	I_Fx	*I_Fo*	*I_diff*	mean of *I_diff* background	p
HTR1	0.058	0.552	-0.494	0.490	0.0002
HTR2	0.058	0.393	-0.252	0.061	0.067
HTR3	0.047	0.844	-0.797	0.126	0.003
HTR4	0.000	0.885	-0.885	0.476	0.001
HTR5	0.797	0.717	0.080	0.126	0.520
