# Supplementary material for: Horizontal transfers between fungal Fusarium species contributed to successive outbreaks of coffee wilt disease
Source: PLoS Biol. 2024 Dec 5;22(12):e3002480. doi: 10.1371/journal.pbio.3002480 (PMC11620798; doi:10.1371/journal.pbio.3002480)
Supplement: S14 Table — Phylogenetic divergence relative to single copy ortholog gene trees. D_Fx = mean pairwise divergence among F. xylarioides genomes for given HTR (substitutions. per site). D_Fo = mean pairwise divergence among F. oxysporum genomes for a given HTR. D_FxFo = minimum pairwise divergence between F. xylarioides HTR and closest F. oxysporum match. p = proportion of values of D_FxFo across single ortholog gene trees ≤ the observed value of D_FxFo for the HTR. Mean D_FxFo across single ortholog gene trees = 0.067, 95% range 0.024–0.153 substitutions per site. (PDF) [file pbio.3002480.s025.pdf]

Table S14: Comparison of divergence patterns for HTR regions to those of background genome regions. Phylogenetic divergence relative to single copy ortholog gene trees
D_Fx = mean pairwise divergence among *F. xylarioides* genomes for given HTR (substitutions per site)
D_Fo = mean pairwise divergence among *F. oxysporum* genomes for a given HTR
D_FxFo = minimum pairwise divergence between *F. xylarioides* HTR and closest *F. oxysporum* match
p = proportion of values of *D_FxFo* across single ortholog gene trees <= the observed value of *D_FxFo* for the HTR
Mean *D_FxFo* across single ortholog gene trees = 0.067, 95% range 0.024-0.153 substitutions per site

	D_Fx	*D_Fo*	*D_FxFo*	*p_FxFo*	*D_FxFFC*	*p_FxFFC*
HTR1	0.001	1.018	0.032	0.094		
HTR2	0.003	0.733	1.410	1.000	1.443	0
HTR3	0.001	0.448	0.018	0.011		
HTR4	0.006	0.121	0.027	0.043		
HTR5	0.080	1.075	0.010	0.001		
HTR6	0.002	1.189	0.015	0.008		
Starship captain	0.000	0.021	0.027	0.001	0.062	0
