# Supplementary material for: Horizontal transfers between fungal Fusarium species contributed to successive outbreaks of coffee wilt disease
Source: PLoS Biol. 2024 Dec 5;22(12):e3002480. doi: 10.1371/journal.pbio.3002480 (PMC11620798; doi:10.1371/journal.pbio.3002480)
Supplement: Attachment — Submitted filename: Reviewer-comments-final.docx [file pbio.3002480.s030.docx]

As you will see in the reviewer reports, while there is recognition of the potential significance of your findings, a significant number of crucial concerns have been raised. Following discussions with the Academic Editor, it is evident that substantial revisions would be necessary to meet the criteria for publication in PLOS Biology. Given our and the reviewers' interest in your study, we are open to considering a comprehensive revision; however, this would require thorough addressing of all reviewers' comments. A successful revision must address issues such as strengthening the HTR hypothesis, correcting the inappropriate use of FST, incorporating available genomic data, including bootstrap support in the phylogenetic analysis, providing support for MIMPS, and enhancing statistical support. Additionally, the revision should clarify the lineage sorting hypothesis and thoroughly discuss it.

>> Many thanks to the editor and reviewers for the helpful comments and suggestions for the manuscript, which have greatly improved the manuscript. We have made a comprehensive revision including several re-analyses and new Figs and tables, which we describe below. Our responses are indicated by >> in blue.

-----------------------------------------
COMMENTS FROM THE ACADEMIC EDITOR:

The critical issues that need addressing, in my opinion, are:

The available genome datasets that weren't used, especially the more comprehensive chromosome-level assemblies and the species that were excluded.

>>As Reviewer #1 suggested, we have re-done our analyses which now rely upon BLAST searches against chromosome-level assemblies and all genomes in the NCBI ‘nr’ database, rather than using our previously downloaded set of *Fusarium* genomes. All Figs have been updated.

The problem of lineage sorting. It's unacceptable for this to be mentioned only once and in an ambiguous manner (page 7), leaving room for this alternative hypothesis without further detail. This needs to be directly tackled (as even reviewer 2 suggested) and discussed more thoroughly in the paper. Personally, I'm skeptical of horizontal gene transfer (HGT/HTR) claims when the boundaries of the transferred segment are undefined.

>>We have performed new analyses and rewritten the discussion to systematically address the evidence for horizontal transfer compared to an alternative hypothesis that they are shared through vertical inheritance (and secondarily lost in the FFC genomes that lack these regions). Overall, the evidence is that 1) the HTR are more similar than expected given the phylogenetic distance between *F. xylarioides* and *F. oxysporum*, 2) the focal genes are in non-homologous locations, 3) they sometimes correspond to mobile elements known to be responsible for horizontal gene transfers in fungi (‘*Starship*’ elements). The new analyses include:

- Sliding window plots showing, for each putative HTR, higher similarity than expected given background levels of sequence divergence between the species (lines 146 to 155 and Fig 3Bi, Fig 3Ci, and S6).
- Bootstrapped gene trees showing closer relationships than expected for each putative HTR between *F. xylarioides* and *F. oxysporum* (with each putative HTR tree evaluated statistically relative to the background genome, measured in the species tree, lines 248 to 257, Fig 3Bii, Fig 3Cii, and S6).
- A synteny plot in new Fig 4 showing that the *F. xylarioides* HTRs match to different locations across accessory chromosomes in *F. oxysporum*, compared to a backdrop of widespread synteny for most of the genome.
- A revised analysis of *Starship* mobile elements in the new Fig 7 showing that HTR 4 corresponds to a *Starship* element that is present only in the arabica population (i.e. missing in the other *F. xylarioides* populations) and with a highly similar “captain” gene to- and overall high sequence similarity to an *F. oxysporum* genome.
- Evidence of multiple well supported clades within the phylogeny of *mimp* transposons that include copies from both *F. xylarioides* and *F. oxysporum*, with the putative HTRs significantly enriched for *mimps*, as well as other classes of TEs which were identified from *F. oxysporum* mobile chromosomes (p<0.001, randomisation test with 1000 trials, see lines 440 to 443).

A full explanation of changes is made against relevant reviewer comments below.

Reviewer 1 expressed several important criticisms, notably regarding the inappropriate use of *F_ST_*.

>>We have removed *F_ST_* from our analyses to solely focus patterns of clustering across a) multiple-locus gene trees and b) SNP variation.

Reviewer 2 criticized the bootstrap support for the phylogenetic trees and the classification into four clades, which is a fundamental issue. Reviewer 3 also highlighted the inadequate bootstrap support, which is really concerning as this is so fundamental to these analyses.

>>In response to Reviewers #2 and #3, we have redrawn all phylogenetic trees with bootstrap branch support values calculated with 100 replicates.

-----------------------------------------
REVIEWER'S COMMENTS:

— — — — — 

Reviewer #1: 

In this manuscript the authors investigate an hypothesis that was proposed previously that there are some regions of the genomes of *F. xylarioides* were potentially as the result of horizontal gene transfer (HTR) from another *Fusarium* species, *F. oxysporum*. To do this, additional genomes have been sequenced, both *F. xylarioides* and *F. oxysporum*. In addition, the authors interrogated whether genes in the putative HTR regions were highly expressed during infection of plants by two of the isolates studied.

>> We thank Reviewer #1 for their helpful comments on the manuscript.

Overall I found that the manuscript seems to consist of two stories, one is about the differences and similarities between *F. xylarioides* isolates, which is in itself interesting and useful with regards to managing these pathogens. The second is the hypothesis that some of genome segments in *F. xylarioides* seem to have originated from another source and share significant similarity with equivalent sequences in *F. oxysporum*.

>>We agree that these are two components, but they are inter-dependent. The observation of differential presence and absence of large genome regions between the different *F. xylarioides* populations can be explained by transfer from *F. oxysporum*. In the revision, we have tried to clarify this connection.

The authors make a convincing argument with regards to HTR, but I miss a number of aspects. The first is that there is a long-read genome for *F. xylarioides* and while they suggest that a full chromosome assembly was not possible, I think that it would be useful to perhaps assign some of the contigs to chromosomes.  Most species in the *Fusarium fujikuroi* species complex have 12 "core" chromosomes and then some have accessory chromosomes. Given that there are chromosome assemblies of some related species this assignation should be possible?

>> We have now aligned our *F. xylarioides* arabica563 reference genome to the *F. verticillioides* chromosomal assembly and renamed our *F. xylarioides* reference contigs accordingly (see dotplot in Fig 1A, Table S13, and lines 86 to 88.

Different strain types featured different putative effector genes, whereby some effectors showed close matches with *F. oxysporum*, but were absent from closer relatives of *F. xylarioides*.  But the authors do not seem to have considered many other species that are in the *Fusarium fujikuroi* species complex - and there are many more genomes available for comparison than those that are used in this study. This would be an easy comparison to make and potentially strengthens the argument for HTR.

>>We originally queried putative effector genes against a set of genomes which included nine representatives from the *F. fujikuroi* species complex (FFC) available at the time of the analysis (see Peck et al. 2021). As recommended, we have updated our results with a BLAST analysis against the entire NCBI ‘nr’ database (results in Fig 3 and S3 Fig) and we still find no blast hits with grade > 50% (where grade combines query coverage, *E*-value and sequence identity values for each hit, see Methods, lines 777 to 781) in any *F. fujikuroi* species genomes for all but one HTR. For example, HTR 2 contains three effector genes which were previously only identified in *F. oxysporum*: *six7*, *six10* and *six12*. These genes yielded no matches in any *F. fujikuroi* species complex genome in our original set, and still yield no matches. A revised Fig 3D shows the phylogeny for all NCBI matches to HTR 2.

It is also not clear why the authors chose to sequence the genomes of *F. oxysporum* isolates in this study (I understand the rationale in terms of which forma specialis were chosen) given that there are many genomes already available including (with one exception) the forma specialis which were chosen for this study.

>> We sequenced the genomes of historic *F. oxysporum* strains that were isolated from coffee wilt disease infected areas in Africa, which we regarded as putative candidate donors for transferred regions following preliminary PCR data which established the presence of our putative effector genes described in Peck et al. (2021) in these strains (see Peck 2023, <https://doi.org/10.25560/103679>). We now explain the rationale for selecting additional isolates in the supplementary methods section “Strain details”.

These other genomes could also be considered in this analysis for the presence of the HTR regions – if only to show that these are not isolate specific in *F. oxysporum*?

>> As a matter of fact, we find a close relationship between *F. xylarioides* HTR 4 / *Starship* and one of these isolates, *F. oxysporum* isolated from a coffee tree (S4C Fig), now highlighted in lines 270 to 272, and 567 to 570. Additionally, *F. oxysporum* f. sp. *pisi* was also clustered with *F. xylarioides* arabica nodes for HTR 5. We hope that the new plots in Fig 3 and S1 Fig show these results more clearly. While we have included the closest *F. oxysporum* BLAST hit in the coverage plots for putative HTRs (Figs 3Bii, 3Cii and S3 Fig), the corresponding phylogenetic trees demonstrate that these hits are not isolate specific in *F. oxysporum.*

An aspect of this study that is discussed is that there are in fact potentially more than one species within the *F. xylarioides* complex.  I think that the authors show good evidence that there are in fact at least two species and to consider these just different populations is confusing and not strictly accurate. I think that this aspect does make understanding this study more complicated.  The fact that the authors "rename" the isolates arabica, robusta and coffea actually speaks volumes to the fact that these are probably three different species.

>> Indeed, there are genetic differences between the populations identified, but we believe that formal description as species would require further evidence, such as mating tests, that are beyond the scope of this study. Also, coffea samples appear to split into two genetic clusters that are just as distinct as the host specialist populations, but we lack geographic or phenotypic evidence to support this. We now highlight in the discussion the need for future work to describe species (see lines 508 to 510).

I do not agree that one can reasonably do an *F_ST_* calculation on such a small number of isolates.

>> We agree and have removed *F_ST_* calculations from the study.

In supplementary Fig 1 the asterisk is not explained?

>>We have removed this Fig.

The names of the genomes and isolates is very complicated, and I found myself getting very confused.

For example, in Figs 3 and 6 the isolate name I039 appears but is not in any of the tables, I had to go back to the previous publication to understand this and then noted that this isolate name is in fact in the supplementary section named as I-394.  Also, there is the *F. verticillioides* isolate that is alternatively 14953 and also 953. The naming's of the isolates is different in supplementary Fig 1 and 2 (for some reason in the supplementary table 2 F. xylarioides 379925 and L0394 are written in italics). I suggest that a single table providing all the names of all the isolates for which genomes have been used in the study is necessary. In addition, the same name needs to be used for a particular isolate throughout the entire manuscript (the supplementary table 2 goes some way to providing this information but then does not include the isolates sequenced in this study). This way when one compares Figs it is possible to easily understand what is going on.

>>We apologise for the confusion caused by our inconsistent strain name and number abbreviations. We have corrected all instances throughout the manuscript and refer to Tables S1 (for strains we sequenced) and S2 (for public genomes) where necessary to direct the reader to the full strain details. For additional clarity, we have re-ordered S2 Table to follow the order of the species tree in Fig 1C.

One of the most perplexing aspects of this manuscript is that in some cases analyses are done using all the genomes and in others the authors have focused only on the genomes that were sequenced in this study.  This is rather unbalanced and does not seem to be needed?

>>With reference to Fig 6 and the analyses of *mimp* transposons, we only used the genomes we sequenced in this study because this required annotation and prediction of repeats and transposable elements, which we did not do for public genomes. However, we used BLAST to look for *mimps* in public genomes and found these mostly in *F. oxysporum* and *F. xylarioides,* with seven *mimp* copies in *F. proliferatum* (see mimp1 maximum likelihood tree in S10 Fig). *F. proliferatum* is a member of the FFC, and a small number of *mimps* were also found in this species by van Dam et al. (2017), ref 31 in the main text (discussed in lines 399 to 401, and 731 to 733). All other analyses use all available genome data.

The last sentence of the abstract states "Our results support the hypothesis that horizontal gene transfers contributed to the repeated emergence of this fungal disease."  This sentence is problematic because I think it refers to *F. xylarioides,* but the last species referred to in the previous sentence is in fact *F. oxysporum*. Another aspect is that while fungi can be causal agents of a plant disease, one cannot strictly refer to them as causing a fungal disease. Surely a fungal disease is a disease of a fungus?

>>We have corrected the abstract to read “Our results support the hypothesis that horizontal gene transfers contributed to the repeated emergence of coffee wilt disease.”

— — — — — 

Reviewer #2: This tour de force by Peck et al seeks to elucidate how horizontal transfers between *Fusarium* species has contributed to the outbreaks of devastating coffee wilt disease. I must commend the authors on such a thorough study, and I must say I enjoy reading it. Therefore, the comments I have seek to improve the overall study and tie up a couple of loose ends.

We thank Reviewer #2 for their helpful comments and are pleased they found the manuscript an enjoyable read.

My main comment on this is that the foundational analyses and findings need a little more support. The follow-on findings will then be bolstered by this. Primarily, the authors state in the Results that 'there are four well resolved groups', yet my issue with this is that there isn't basic bootstrap support on the phylogenetic analysis to support this. Then, when talking about coffea strains falling into two distinct clades, again, bootstrap support would help here. For these two clades it would be worth noting what makes them distinct…

>> Apologies for the omission. We have rewritten the result section “*Fusarium xylarioides* is a monophyletic species complex with genetically differentiated populations”. *Fusarium xylarioides* forms a monophyletic clade within the *F. fujikuroi* species complex, a result that receives 100% bootstrap branch support in concatenated analysis of single-copy genes (1000 replicates, Fig 1C) and 58% support in the "supergene" tree (branch support values, S2 Fig). While relationships were not highly congruent in the “supergene” tree for the *F. xylarioides* populations, we show that in the “supermatrix” tree (Fig 1C and the new S1 Fig, a zoom on the *F. xylarioides* clade) all the clades we discuss have 100% bootstrap support in the concatenated tree. This is now referred to in the text in lines 143 to 146, and 152 to 158.

Fig 2 and our Neighbour-Net analysis then supports the existence of genetically differentiated populations with only small amounts of reticulation between some pairs of populations (lines 175 to 183).

… i.e. are there differences in gene content (this may have been done and I didn't see it, in which case, make it a bit clearer).

>>Across all orthogroups that are present in *F. xylarioides*, 80% genes are shared by all 4 populations (lines 154 to 158 and new S2 Fig), and we discuss some of the gene content which differs, the putative effector genes, in later sections.

Perhaps my biggest comments are:
- does temporal analysis using BEAST/BEAST2 confirm your findings? This may be beyond the scope of this study, but would be nice.

>> Thanks for the interesting idea, but we feel a BEAST analysis is beyond the scope of this study as our focal questions do not consider timings of divergence.

- I think there needs to be a bit more consideration of recombination and/or gene flow, which would be resolved by including an ADMIXTURE and/or fineSTRUCTURE analysis. Indeed, the latter would help resolve ancestral donation and inferring the 'donor strains'.

>>Similarly to Reviewer #1 comments about *F_ST_*, we think that an ADMIXTURE approach could be interesting but only with a larger sample of isolates from each population; and therefore decided not to add it here but leave it for future work.

Minor comments:

Data availability: for me, I get the notification that there's been no data deposited or its awaiting submission/validation.

>>The reads are now available through the Sequence Read Archive under the accession PRJNA1043203. The genome assemblies are submitted under the same BioProject and awaiting validation.

Introduction: a couple of sentences describing the yield losses (amounts/percentage) attributed to fungi would help non-*Fusarium*/fungal specialist readers. Also, perhaps expanding the recent disease severity in Ethiopia. Is this due to climate change?

>> We have added estimates of losses in yield due to fungal diseases to the opening sentences (10-23%). With regards to the recent disease severity in Ethiopia, there is unfortunately not enough evidence to understand what is driving the increase. Subsequently, we have clarified this level of uncertainty with the statement “However, one recent study suggests that disease severity in Ethiopia has increased (Mulatu and Shanko, 2019, ref 14 in the main text)” (lines 69-70).

Also in the introduction, it should be noted that *Starships* haven't been proven to be involved in HGT (i.e. no one has 'seen' them move) - see Bucknell et al 2023 Mol Microbiology. At the moment, it's all sequence evidence, which is fine, but we are not completely 100% sure of how they are moving.

>>With regards to *Starships*, there is to date one study with experimental evidence of a *Starship* moving in the mould *Paecilomyces variotii*: a strain was created with an antibiotic resistance cassette disrupted with a *Starship*, in 13/15 strains tested the *Starship* reintegrated elsewhere within the genome of these strains, in which antibiotic resistance was maintained (Urquhart et al., 2023, ref 20 in the main text). We have added an additional line (line 82-83) describing this “In addition to genomic evidence, there has been experimental evidence of Starships as autonomous transposons (Urquhart et al., 2023, ref 20 in the main text).

Methods: 'other *Fusarium* genomes' - more details needed in the text on the sequence read archive or refer to Table 1.

>>As suggested, we have included a reference to Table 1 “RNA-Seq reads for the closest *formae speciales* match were downloaded from the sequence read archive (see Table 1 for accession numbers) and aligned to the corresponding assembly using ‘bbmap.sh’)”.

Is the TE/repeat annotation library available for community use?

>>Yes, this is uploaded to a github repository. We reference this on lines 725-726.

Importantly, how were orthologous gene group trees rooted (needs mention in Methods)?

>> The STRIDE algorithm identiﬁes sets of well-supported in-group gene duplication events from a set of unrooted gene trees and analyses these events to infer a probability distribution over an unrooted species tree for the location of its root. We have included an additional sentence in the methods describing how OrthoFinder infers the rooted species tree: “OrthoFinder infers gene trees for all orthogroups and uses gene duplication events via the STRIDE algorithm to root them (Emms and Kelly, 2019)” (see lines 743 - 744).

How does your *Starship* identification align with the 'official' starfish method for discovering these?

>>We have now redone the *Starship* identification to use the recently published ‘starfish’ pipeline (Gluck-Thaler and Vogan, 2023, ref 83 in the main text), and updated the methods and results sections to reflect this (lines 799 to 813). As a note, starfish focuses upon identifying clean cases of insertion and is therefore very conservative in approach, as it can miss insertions in complex regions such as bordering structural variants or assembly breaks.

Figs: a description of what the scale is describing is needed in the legend for all phylogenies.

>>We have added the statement “The scale bar indicates branch length (XX substitutions per site)” (where XX is the length of the scale bar in the figure) to each Fig legend where the Fig includes a phylogenetic tree with a scale bar.

I couldn't find Table S1!

>>We apologise, the order of supplementary Figs and tables was mixed up, this has now been corrected.


— — — — — 

Reviewer #3: The manuscript by Peck et al. analyzes the outbreaks caused by the coffee wilt disease agent *Fusarium xylarioides*. The authors take advantage of historical samples for a total of 13 strains to investigate the genetic structure and host associations. The authors indicate that virulence was acquired at least in part through horizontal transfer of effector genes. Roles in virulence were supported by transcriptomic analyses during infection.

The manuscript reports additional genomes for *F. xylarioides* over existing historical genome datasets and provides new dimensions of data analyses. However, much of the major claims of horizontal gene transfer and virulence components are not supported by robust analyses allowing for hypothesis testing. Hence, most of the manuscript remains largely descriptive and suggestive. The main claim stated in the title and elsewhere that horizontal transfer contributed to outbreaks, even successive outbreaks, lacks support on multiple levels.

>> We thank the reviewer for their helpful comments and suggestions. We have added new analyses and statistical tests to add support to the evidence for horizontal transfer which we hope addresses their concerns, outlined below.

Evidence for HTRs: the authors describe "putative HTRs" by identifying similar genomic regions between *F. oxysporum* and *F. xylarioides*. I am struggling to understand how the evidence for this was evaluated. Neither the main results nor Fig 4 provide direct support. Trees should include bootstrap values.

>> Substantial improvements have been made to the HTR hypothesis with additional analyses and new Figs. The key new parts are:

1) We now plot scaffolds of each HTR showing mean similarity between the arabica population and *i*) the robusta and coffea populations one the one hand, and *ii*) the closest *F. oxysporum* match from the NCBI ‘nr’ database or our newly sequenced genomes on the other hand (Figs 3C–D, S1A–D)*.* We show that these regions display significantly higher genetic similarity to *F. oxysporum* than to the other *F. xylarioides* populations: “For HTRs 1 to 4, the mean sequence identity to closest matches in other *F. xylarioides* strains (denoted *I_Fx*) was 5.8%, 5.8%, 4.7% and 0% respectively, whereas the mean identity to closest *F. oxysporum* matches (*I_Fo*) was 55.2%, 39.3%, 84.4% and 88.5%. To test if these values were statistical outliers compared to the genome-wide average, we aligned similarly sized windows to both *F. xylarioides* and *F. oxysporum* genomes and compared the observed values of the difference (*I_diff* = *I_Fx - I_Fo*) to this background distribution. The proportion of background windows with an *I_diff* < the observed value was 0.018%, 6.7%, 0.25% and 0.54% for HTRs 1 to 4 respectively, indicating that most HTR regions were extreme outliers compared to the background distribution of sequence identity across *F. xylarioides* and *F. oxysporum* genomes S13 Table)”. This is reported in lines 248 to 257.

2) We now show phylogenetic trees for alignments of each whole HTR region including our samples and all BLAST hits from the NCBI ‘nr’ database with bootstrap values (Figs 3C–D, S1A–D). We show that the pairwise sequence divergence between *F. xylarioides* and the closest *F. oxysporum* match is significantly lower than background levels across the genome: “To test if pairwise divergence between the putative HTRs and the nearest *F. oxysporum* (*D FxFo*) copy were statistical outliers compared to background levels of divergence, we compared putative HTR gene trees with the nearest *F. oxysporum* copy (*D FxFo*), and then compared the observed values of the difference with the metric of divergence in single-copy gene trees. The proportion of HTRs with a *D FxFo <*= the observed value was: HTR 1, 9.4%; HTR 3, 1.1%; HTR 4, 4.3%; HTR 5, 0.13%; HTR 6, 0.84%, S14 Table. In HTR 2, the same genome region was recovered from one or more *F. fujikori* complex species, but they clustered with different *F. oxypsorum* copies in the tree (Figs 3C and S5A-B). Pairwise divergence between the *F. xylarioides* and closest *F. oxysporum* match (*D FxFo*) was unexpectedly lower than the divergence between *F. xylarioides* and the closest species from the *F. fujikori* complex species match (*D FxFFC*), relative to the expected levels (percentile of *F FxFo < D FxFFC*) across single copy ortholog trees: 0.0%).” This is reported in lines 273 to 284.

3) A new Fig 4 plots synteny between the *F. xylarioides* arabica reference genome and two *F. oxysporum* genomes, highlighting that putative HTRs show their close matches to multiple *F. oxysporum* accessory chromosomes.

4) We now highlight in the discussion (lines 511 to 531) all the pieces of evidence for HTRs, namely their complete lack in other populations and closely related species (see Figs 3C–D and S3), their different genomic locations across *F. xylarioides* host-specific populations (for example, HTR 2 in arabica and coffea strains and HTR 6 in robusta and coffea strains, which match to different parts of the same region of an *F. oxysporum* mobile pathogenic chromosome) and the existence of a *Starship* mobile element present in the arabica strains only.

As *F. oxysporum* and *F. xylarioides* are closely related, how was the alternative explanation of incomplete lineage sorting evaluated?

>>We have now included a more extensive discussion of how we evaluated alternative hypotheses to explain our putative HTRs (see lines 511 to 531). Briefly, most of the putative HTRs are present in the arabica population of *F. xylarioides* and in *F. oxysporum*, but absent from other *F. xylarioides* populations or any *F. fujikuroi* species complex (FFC, which includes many phylogenetically closer relatives of *F. xylarioides*) genomes present in the NCBI ‘nr’ database. So, an alternative hypothesis to horizontal transfers would be that the region was present in the common ancestor of *F. oxysporum* plus the *F. fujikuroi* species complex, but subsequently lost in all the sampled *F. fujikuroi* species complex genomes except the arabica population of *F. xylarioides*. This could be phrased as incomplete lineage sorting if the presence/absence of the HTR region was polymorphic in the common ancestor. We evaluate that the hypothesis of shared vertical inheritance is not well supported, however, because:

1) The shape of the species tree (Fig 1C) would require six independent loss events to explain the pattern of presence/absence of the focal region across strains, which seems unlikely.

2) The new analyses described above show that the sequence similarity between *F. xylarioides* HTRs and their closest *F. oxysporum* matches are significantly higher than would be expected based on background genome levels (Fig 3C and D, S3 A–C Fig): “For HTRs 1 to 4, the mean sequence identity to closest matches in other *F. xylarioides* strains (denoted *I_Fx*) was 5.8%, 5.8%, 4.7% and 0% respectively, whereas the mean identity to closest *F. oxysporum* matches (*I_Fo*) was 55.2%, 39.3%, 84.4% and 88.5%. To test if these values were statistical outliers compared to the genome-wide average, we aligned similarly sized windows to both *F. xylarioides* and *F. oxysporum* genomes and compared the observed values of the difference (*I_diff = I_Fx - I_Fo*) to this background distribution. The proportion of background windows with an *I_diff* < the observed value was 0.018%, 6.7%, 0.25% and 0.54% for HTRs 1 to 4 respectively, indicating that most HTR regions were extreme outliers compared to the background distribution of sequence identity across *F. xylarioides* and *F. oxysporum* genomes (S13 Table).” This supports recent horizontal transfer, whereas the vertical inheritance hypothesis would predict that sequence divergence should fall within the variation observed across the rest of the genome.

3) Fig 4 now shows that the putative HTR regions are located at different genomic positions in the arabica reference genome compared to *F. oxysporum*. Given the high degree of synteny observed between these species across the rest of the genome (as shown by grey blocks in Fig 4), these regions might also be expected to fall into syntenic blocks if inherited vertically from a common ancestor.

4) We have found a *Starship* element exactly corresponding with an HTR, and *Starship* elements have been repeatedly involved in horizontal gene transfers between fungal species (e.g., see Gluck-Thaler et al., 2022, ref 18 in main text; Urquhart et al., 2023, ref 20 in main text; McDonald et al., 2019, ref 16 in main text).

The results should also more clearly state how cut-offs for similarity were defined using genome-wide average divergence,

>> Further details on similarity cut-offs are provided on lines 763 to 776, and 777 to 783. Briefly, similarity between *F. xylarioides* arabica563 and all other genomes was analysed using BLASTn to query all 10 Kb regions of the genome (including coding and non-coding DNA) in the nr database. We recovered the top 50 hits with a grade > 50%, or all hits > 50% if there are fewer than 50 hits (grade combines query coverage, e-value and identity values for each hit to long, high identity hits). We have also added a new Methods section, “Statistical evaluation of putative horizontal transfers” which describes our tests to identify sequence identity outliers (see lines 814 to 835).

i.e. how much more similar are putative HTRs compared to the genome-wide average divergence?

>>We now include results reporting similarity relative to genome-wide average (lines 248 to 257, S13 Table).

Was similarity restricted to coding sequences or did similarity expand across the entire HTR including non-coding sequences?

>>We now search entire regions, so this includes both coding and non-coding sequences, although in some parts of the results we also focus on genes of interest within the HTR regions such as the SIX genes.

The issue of similarity analyses extends also to the Six gene analyses where it remains unclear whether the reported similarities are true outliers compared to expectations. Six genes are expected to diverge more rapidly. What divergence or similarity is hence considered "unusual", falling outside of the expectation for vertical inheritance?

>>We have updated our HTR analyses to include phylogenetic trees of all top BLAST hits for each putative HTR. *F. xylarioides* copies of HTR 2 (which contains the three SIX genes) clusters with the *F. oxysporum* species complex, and the SIX genes themselves only have positive blast matches to *F. oxysporum* genomes. The alternative hypothesis would be that the HTRs were acquired by the common ancestor of *F. xylarioides* and *F. oxysporum*, and lost from every other member of the *F. Fujikuroi* species complex. However, due to the shape of the species tree (Fig 1C) this would have required six independent loss events, which seems unlikely.

Also, for HTR 4, the authors report 88% flanking sequence identity, 90% genome-wide identity and >94% defined as "high" identity. Are these true outliers / what percentiles?

>>The percentiles for the observed similarities of each HTR are now reported in S14 Table, as described above.

Do *Starship* regions suspected to be HTR share higher similarity than other regions? Can you provide more insights into the "overlap" of a *Starship* and HTR3?

>>We have reannotated *Starships* using the starfish pipeline, and now demonstrate that HTR 4 does indeed correspond to an annotated *Starship*. We have rewritten this section and added a new Fig 7 to improve the clarity of this section. We have included a test showing that the pairwise divergence between the arabica *Starship* and *F. oxysporum* *Starship* is in the lower 0.1 percentile of genome baseline (lines 468 to 469, S14 Table).

What is the suspected mechanism? That the Starship was the mechanism of HT?

>>We do not have evidence for mechanism here, but *Starships* have been implicated in horizontal transfer in other systems, such as the *ToxA* gene transfer between wheat pathogens, and mobility has been demonstrated by Urquhart et al. (2023, ref 20 in the main text) in another system. It is therefore a potential mechanism for horizontal transfer, but more work would be needed to test this. We have revised the discussion of this point to clarify (see lines 522 to 524).

Overall, without proper statistical support, it is difficult to discussing evidence for HTR in this system.

>> We hope our new tests described above and revised discussion of the evidence suitably address this concern.

Upregulation of CAZymes and predicted effectors: This section suggests that there is an overlap between gene upregulation and candidate genes involved in virulence. I lack though a clear conclusion from the section. Was the aim to show that effector candidates tend to be upregulated in planta? Were effector candidates more likely to be upregulated? Furthermore, how is the statement "It appears that a subset of CAZyme families have been acquired..." related to the HTR section above? What HTR region is this? How strong is the evidence for HT there? Is the pattern of upregulation conserved among the homologous segments in the putative donor and recipient strain genomes?

>>This section previously had two parts: i) that effector candidates, including those on HTRs, were indeed upregulated in planta during infection (Fig 5B); ii) and, more broadly, that expressed CAZyme genes were phylogenetically closer to various *F. oxysporum* strains than the single-copy genes in the species tree. To simplify this section, we have removed the second part to just focus on the upregulated effector candidates, especially genes that are putatively shared between *F. xylarioides* arabica and *F. oxysporum* isolates.

Analyses of *miniature impala* elements and HTR: Here I also lack a clear statistical support whether e.g. *mimps* are more likely to be putatively horizontally transferred. The authors simply refer to the fact that some *mimps* were found in HTRs. 
Is "shared" TEs between *F. xylarioides* and *F. oxysporum* to be understood as being horizontally transferred or shared by recent, common descent? What statistical analyses support either of these interpretations?

>> We have rewritten this section to convey that *mimps* are significantly enriched in the putative HTR regions (shown with a new randomisation test: “probability of observing as many or more in randomly selected 44 10 Kb windows from the genome p < 0.001 (1000 trials)”), and absent in other members of the *F. fujikuroi* species complex, except *F. proliferatum* (see lines 399 to 401, 440 to 443, and 731 to 733). Our tests for HTR per se primarily focus on HTRs and starship, whereas *mimps* are of interest because of their association with HTRs and effector genes in *F. oxysporum*. Our wording now reflects this more clearly (e.g. heading for *mimp* section).

Additional comments:
- Fig 3B: The authors attribute the small amount of reticulation in the network to gene flow. However, this could be explained by a lack of resolution of the network and/or historic recombination. Later this possibility is mentioned. Clarifying the reticulation pattern would be helpful.

>>We have revised the discussion on this point on lines 180 to 183.

- *F_ST_* analyses among genetic groups seem inappropriate as it remains unclear whether each group indeed consists of a population of interbreeding individuals (a requirement for calculating *F_ST_*). A group size of ~2-4 haploid individuals is also inappropriate to estimate allele frequencies (necessary for *F_ST_* calculations).

>> We concur and have removed *F_ST_* analyses to focus on other approaches.

- Discussing the existence of "populations" with such small group sizes of genetic clusters seems tenuous. Without further sampling efforts, terms like "lineage" or "genetic cluster" seem more appropriate.

>>The arabica and robusta populations have been reported by earlier studies sampling more isolates and conducting marker-based sequencing and phenotyping including host specialisation (see Lepoint 2006 and Girma 2004, refs 37 and 38 in the main text). We agree that the newly discovered clusters in the coffea samples have no independent information to inform on their status, but the level of divergence is similar to that observed between the arabica and robusta populations. Combined with evidence from the SplitsTree for their relative isolation from the others, we call them ‘populations’ as well for simplicity but note that more sampling would be required to identify the status of the two coffea clusters (see lines 507 to 510).

- The Circos plot identifies identities spanning 0-1. What is the interpretation of similarities of e.g. 0.2? Isn't 20% similarity at the level of random expectation for sequence similarity? Similarities would typically only be reported for syntenic or aligned regions. So, 70-80% might be the lower limit for this. Clarifying this in the legend would be helpful.

>>We have updated this Fig to remove the Circos plot and instead show a line plot of nucleotide similarity calculated in 10 kb windows, with the average value shown for each *F. xylarioides* population. Low values in the new Fig 3A are possible because:

The genomes were each aligned to the arabica563 reference using Minimap2. Alignment similarity was calculated in contiguous 10 kb windows, with similarity in each window calculated per 100 bp with no slide using ‘bedtools coverage’. Mean similarity was calculated for each population group (arabica, robusta, coffea1 and coffea2) for each 10 kb window. So, a region with no match (as detected by bedtools coverage) would get a value of 0, but the wider 10kb window may also contain a positive match in which case Fig 3A can show any value between 0 and 100%.

We have clarified the approach taken in the methods, lines 763 to 776.

— — — — — 

Reviewer #4: This manuscript "Horizontal transfers between fungal *Fusarium* species contributed to successive outbreaks of coffee wilt disease" investigate the coffee wilt disease caused by the fungal pathogen *Fusarium xylarioide*s focusing on the sub-Saharan Africa area. Authors sequenced and compared 13 genomes, including 11 *F. xylarioides*, *F. oxysporum* and *F. solani*, generated one *F. xylarioides* genome using long-read to be used as the *F. xylarioides* reference genome, and conducted comparative genomics and transcriptomics analysis. This is an important research topic. 

Authors have done tremendous work, trying to answer at least 3 rather complex biological questions: 1) populations structure of *F. xylarioides* isolates 2) HGT, and 3) mechanisms of HGT. Unfortunately, I am not able to derive the same conclusions authors presented here based on the data presented. I believe more careful analysis that testing specific hypothesis proposed in the manuscript is necessary.

>> We thank Reviewer #4 for their appraisal of the work and their helpful comments below.

Specific comments. 

First, authors concluded that *F. xylarioides* comprises at least four distinct lineages: one host-specific to Coffea arabica, one to *C. canephora* var. *robusta*, and two historic lineages isolated from various Coffea species. The data presented in the manuscript doesn't support this. 

* Fig 2a supports the monophyletic nature of the *F. xylarioides* isolates. 2b shows no differences among *F. xylarioides* genomes. Current phylogenetic analyses, including Fig 3 didn't provide strong evidence supporting the distinct affiliation with different coffee hosts.

>>We have rewritten the result section “*Fusarium xylarioides* is a monophyletic species complex with genetically differentiated populations”. *Fusarium xylarioides* forms a monophyletic clade within the *F. fujikuroi* species complex, a result that receives 100% bootstrap branch support in concatenated analysis of single-copy genes (1000 replicates, Fig 1C) and 58% support in the "supergene" tree (branch support values, S2 Fig). While relationships were not highly congruent in the “supergene” tree for the *F. xylarioides* populations, we show that in the “supermatrix” tree (Fig 1C and the new S1 Fig, a zoom on the *F. xylarioides* clade) all the clades we discuss have 100% bootstrap support in the concatenated tree. This is now referred to in the text in lines 93 to 99, and 105 to 110.

* In addition, Fig 2 should use the single-copy genes instead of genes have members in all genomes. Bootstrap values are needed on the phylogeny.

>> Based on the Reviewer’s suggestion, we now present a “supermatrix tree” reconstructed from a concatenated alignment of all single-copy genes with bootstrapping (see Fig 1) including a zoom on the *F. xylarioides* clade (see S1 Fig). We have moved the default STAG species tree of OrthoFinder, or “supergene tree”, to new S2 Fig, which uses genes with at least one copy in all genomes, as the multi-copy gene trees are as accurate as single-copy trees but with an 8-fold higher data utilisation (see <https://www.biorxiv.org/content/10.1101/267914v1>). Support values for bipartitions on this tree are the proportion of gene trees that contain that bipartition. The topology of the two trees are the same.

* Also, a detailed table capture all *F. xylarioides* strains included in the analysis, not only sequenced strains, may help to see the relationships among these strains from both geological and historical perspectives.

We have added strain information in S1 Table (strains sequenced in this study) and S2 Table (public strains used in this study).

Second: authors concluded that host-specificity appears to be acquired through horizontal transfer of effector genes from members of the *F. oxysporum* species complex by comparing a *F. oxysporum* tomato pathogen to the *F. xylarioides* coffee pathogen and identified regions on contigs 3, 6, 10, 11 and 13. 
* Why not the *F. oxysporum* coffee pathogen?

>>The *F. oxysporum* coffea pathogen is a relatively minor pathogen which acts in conjunction with the nematode *Meloidogyne incognita* on *Coffea arabica* in the Americas, not Africa (Bertrand et al., 2000, Lopez-Lime et al., 2020, Negron and Acosta, 1989; see below for references). Because there has presumably been limited opportunity for contact with *F. xylarioides* in Africa, we did not focus our studies on that strain. We do, however, include an *F. oxysporum* strain which was isolated from *Coffea arabica* in Tanzania. With formal species identification of this strain lacking, and in consideration of the above, we do not refer to it as *F. oxysporum* f. sp. *coffea.*

Instead, we chose *F. oxysporum lycopersici* (from tomatoes) because *F. xylarioides* has been isolated from tomatoes in Nigeria (see Onesirosan et al., 1976, details below) and there may be opportunities for the two fungi to occupy similar niches in *Solanaceous* weeds in the coffee gardens.

Bertrand, B., Nunez, C. and Sarah, J.L., 2000. Disease complex in coffee involving Meloidogyne arabicida and Fusarium oxysporum. *Plant pathology*, *49*(3), pp.383-388.

López-Lima, D., Carrión, G., Sánchez-Nava, P., Desgarennes, D. and Villain, L., 2020. Fungal diversity and Fusarium oxysporum pathogenicity associeted with coffee corky-root disease in Mexico. *Revista de la Facultad de Ciencias Agrarias*, *52*(1), pp.276-292.

Negron, J.A. and Acosta, N., 1989. The Fusarium oxysporum f. sp. coffeae-Meloidogyne incognita complex in'Bourbon'coffee. *Nematropica*, pp.161-168.

Onesirosan, P. T., & Fatunla, T. (1976). Fungal Fruit Rots of Tomatoes in Southern Nigeria. *Journal of Horticultural Science*, *51*(4), 473–479.

* As presented in Fig 1 and stated in the manuscript, "HTRs were differentially present across the *F. xylarioides* populations". If these regions are important for *F. xylarioides* pathogenicity to coffee, why they are so different among different *F. xylarioides* isolates?

>>We hypothesise that these are important for host specialisation on different coffee varieties rather than for pathogenicity on coffee per se. We have clarified this point in the discussion, see lines 398 to 403.

* Do these present/absent polymorphisms have any relationship to the *F. xylarioides* population structure?

>>Yes, we emphasise this further now with a new section highlighting differential presence/absence of genome regions in the different *F. xylarioides* populations (see line 131), before discussing the evidence that these result from HTR. We also present a new Fig (S2 Fig) that shows gene presence/absence among populations as a Venn diagram.

Third, authors presented the affiliation of *mimps* between *F. oxysporum* genome and *F. xylarioides* and concluded that facilitate HGT. Turning affiliation into facilitation is too big a leap.

>>Yes, we agree that the causal role of *mimps* in transfer is hypothetical at the present. We have reworded carefully (e.g. lines, 376 to 378).

Overall, authors added unsupported speculation in the discussion section. For instance, authors claim that "the *F. xylarioides* formae speciales proposed nearly two decades ago can now be recognised as genetically differentiated populations with different host-specificity". In the result section, authors stated that there are 4 distinct populations. Then which strains will be classified as *F. xylarioides* f. sp. *abyssiniae* and *F. xylarioides* f. sp. *canephorae*, respectively?

>>As outlined above, we do not think that we can reclassify anything with our data here, and so we have removed this section and we refer to the four groups as populations, of which two represent the previously well-supported arabica and robusta host forms.
